# MEX3C interacts with adaptor-related protein complex 2 and involves in miR-451a exosomal sorting

**DOI:** 10.1371/journal.pone.0185992

**Published:** 2017-10-05

**Authors:** Pin Lu, Huanhuan Li, Ning Li, Ravi N. Singh, Colin E. Bishop, Xiangxian Chen, Baisong Lu

**Affiliations:** 1 Anhui Normal University, Wuhu, China; 2 Institute for Regenerative Medicine, Wake Forest University Health Sciences, Winston Salem, North Carolina, United States of America; 3 Department of Cancer Biology, Wake Forest University Health Sciences, Winston-Salem, North Carolina, United States of America; George Mason University, UNITED STATES

## Abstract

Some RNA species, especially microRNAs, are non-randomly sorted into exosomes, but how selectivity of RNA exosomal sorting is achieved is unknown. We found that all three variants of RNA-binding ubiquitin E3 ligase (MEX3C)–MEX3C-1, MEX3C-2, and MEX3C-3 –interact with adaptor-related protein complex 2 (AP-2), a cargo adaptor in clathrin-mediated endocytosis. MEX3C’s C-terminal RING finger domain and the hnRNP K homology (KH) domain shared by the three MEX3C variants are both necessary for MEX3C/AP-2 interaction. MEX3C associates with the endolysosomal compartment through an endocytosis-like process. siRNA-mediated inhibition of the MEX3C or AP-2 complex substantially decreased exosomal but not cellular microRNA miR-451a expression. Exosomal sorting is ceramide-dependent but not ESCRT-dependent in microRNA miR-451a. That RNA-binding protein associates with membrane trafficking machinery, and that its involvement in exosomal microRNA expression, suggest the existence of a mechanism for specific recruiting of RNA molecules to endosomes for subsequent exosomal sorting.

## Introduction

Extracellular vesicles (EVs) contain RNA species such as microRNAs (miRNA), and may mediate intercellular RNA transfer [1]. Some miRNA species, including miR-451a, are selectively enriched in EVs [2]. Consistent with the observation that ceramide triggers budding of exosome vesicles into multivesicular endosomes [3], miRNAs can be sorted to the exosomes through a ceramide-dependent mechanism [4]. The Endosomal Complexes Required for Transport (ESCRT) complexes are also involved in exosome biogenesis [5], although whether they play a role in miRNA exosomal sorting is unclear. Some miRNAs contain sequence motifs to guide their EV sorting [6,7]. On the hand, RNA-binding proteins, such as SYNCRIP, hnRNPA2B1, and Y-box protein 1, mediate the exosomal sorting of miRNAs [8–10]. However, the link between ribonucleoprotein particles and membrane vesicles is unknown.

Adaptor protein-related complex 2 (AP-2) plays an important role in clathrin-coated vesicle-mediated trafficking [11,12]. It possess four subunits, including a small subunit σ2 (encoded by *AP2S1*), a medium-sized subunit μ2 (encoded by *AP2M1*), and two large subunits, α (encoded by *AP2A1* or *AP2A2*) and β2 (encoded by *AP2B1*). AP-2 primarily locates on the plasma membrane as a cargo adaptor and facilitates clathrin assembly during endocytosis [11,12]. It may have unappreciated functions beyond endocytosis, since it is also observed on endosomes [13–15] and mature lysosomes [16], and inhibits AP-2 expression affects post-endocytic trafficking [17].

AP-2 recognizes sorting signals on the membrane proteins to be internalized. These include tyrosine-based (YXXΦ) and dileucine-based [(D/E)XXXL(L/I)] sorting signals (X indicates any amino acid, Φ indicates bulky hydrophobic amino acid, other letters are single letter amino acid codes) [18–20]. Ubiquitination is also an internalization signal for membrane proteins. AP-2 and ubiquitin adaptor proteins such as EPSIN and EPS15 are needed for internalization of ubiquitinated membrane proteins. EPSIN and EPS15 interact with each other [21]. Both proteins interact with AP-2 [21–23] and contain ubiquitin interacting motifs to bind ubiquitin chains [24]. However, little is known about how non-membranous cytosolic proteins are recruited to the endosome system.

MEX3C is an RNA-binding ubiquitin E3 ligase. It has two hnRNP K homology (KH) domains with single-strand RNA binding activity [25], and a C-terminal ring finger domain with ubiquitin E3 ligase activity [26,27]. At least three MEX3C protein variants can be expressed by alternative splicing and alternative transcription initiation [28]. The longest MEX3C variant, human MEX3C^659AA^ (containing 659 amino acid residues), shuttles between the nucleus and the cytoplasm due to the presence of nuclear export and nuclear localization signals [25], and functions as an adaptor for exportin 1-mediated *FOS* mRNA nuclear export [28]. This long and shuttling MEX3C isoform is termed MEX3C-1 in this manuscript for clarity and simplicity.

MEX3C-1 is the mostly investigated variant so far. It transfers K63-linked ubiquitin chains to RIG-I to enhances RIG-I–mediated antiviral innate immunity [27], and to CNOT7 to facilitate the degradation of *MHC-I* mRNA [29]. The two shorter MEX3C variants, MEX3C^464AA^ and MEX3C^372AA^ (containing 464 and 372 amino acid residues, respectively) [28], are named MEX3C-2 and MEX3C-3, respectively, in this paper. Although little is known about the physiologic functions of MEX3C-2, mRNAs coding for this variant are most widely expressed [28]. Human *MEX3C* contributes to genetic susceptibility to hypertension [30] and represses chromosomal instability of tumor cells [31]. Mouse *Mex3c* is involved in regulating postnatal growth [32] and energy expenditure [33,34], although the mechanisms remain unknown.

While searching for MEX3C-interacting proteins through affinity purification and mass spectrometry, we unexpectedly observed the α and β2 subunits of AP-2 complex among MEX3C-associated proteins. Here we describe the interaction of MEX3C with the AP-2 complex, and the involvement of MEX3C in sorting microRNA miR-451a to exosomes. Our findings provide evidence as to a potential mechanism for selective miRNA sorting into exosomes.

## Materials and methods

### Chemicals

Neutral sphingomyelinase 2 inhibitor GW 4869 (hydrochloride hydrate) was purchased from Cayman Chemical Co. (Item no. 13127). Puromycin and Bafilomycin A1 were purchased from Sigma.

### Plasmids

Plasmid DNA constructs for the expression of Flag-tagged human MEX3C-1 (pFlag-MEX3C-1), KH domains-deleted mutant MEX3C-1ΔKH (pFlag-MEX3C-1ΔKH), C-terminal 413–659 AA deleted mutant MEX3C-1ΔC (pFlag-MEX3C-1ΔC), mouse MEX3C-2 (pFlag-MEX3C-2), and mouse MEX3C-3 (pFlag-MEX3C-3) have been described previously [28]. The other plasmids used in the study are described in Table A in Supplemental S1 File. All the constructs created in our laboratory were verified by sequencing.

### Searching for MEX3C-associating proteins by affinity purification and mass spectrometry analysis

Flag-tagged mouse MEX3C-2 (different from human MEX3C-2 by three amino acid residues) was transiently expressed in HEK293 (ATCC, Manassas, Virginia) by Fugene 6 (Roche)-mediated transfection of pFlag-MEX3C-2 plasmid DNA. Cells from five 15-cm dishes with or without MEX3C-2-Flag expression were used for the protein complex purification. Cells were lysed with 10 ml NP-40 buffer and 0.5 ml anti-Flag agarose beads (Sigma) was added to each pre-cleared lysate for the affinity purification. After washing with 10 ml NP-40 lysis buffer, the protein bound to the beads was eluted with ten 0.5 ml aliquots of 0.1 M glycine HCl (pH 2.5). The presence of MEX3C-2 in the eluent was examined by SDS-PAGE followed by Western blotting analysis with an anti-Flag antibody (Sigma). Flag-tag positive fractions from MEX3C-2 expressed preparation were pooled. Corresponding fractions from control preparations were similarly pooled. The two samples were concentrated to the same volume by spinning the samples in Centricon 10 (Millipore). The proteins in the samples were separated on an 8~16% gradient Ready-gel (Bio-Rad) and stained with Coomassie blue. Bands unique to the MEX3C-2-Flag preparation were recovered from the gel, treated with trypsin, and subjected to LC-MS/MS analysis to identify the identities of the proteins (analyzed by the Biomolecular Resource Facility of Wake Forest University).

### Immunoprecipitation

HEK293T cells (ATCC) transfected with various expressing constructs were lysed with NP-40 lysis buffer (for analysis of MEX3C-associated RNA) or RIPA buffer (for MEX3C/AP-2 interaction) with protease inhibitors (0.5 mM PMSF and 1x Complete Protease Inhibitor Cocktail; Roche) and phosphatase inhibitors (50 mM NaF, 1.5 mM Na_3_VO_3_). The lysates were centrifuged at 4°C at 12000 g for 15 min and the cleared lysates were pre-cleared with 30 μl protein A/G beads (Santa Cluz). About 1 μg antibody was added to the cleared supernatant and incubated at 4°C for 1 hr. When RNase treatment was performed, 100 μg/ml RNase A was added to the lysates and the lysates were kept at room temperature for 30 min before the addition of antibodies. One hour after antibody addition, 30 μl of protein A/G agarose beads was added and incubated for another 30 mins. Following 3 washes with the respective buffers, the beads were either lysed in 50 μl of 1xSDS loading buffer for SDS-PAGE and Western blotting analysis, or used for RNA extraction using the miRNeasy kit (Qiagen).

### Antibodies used for Western blotting analysis and immunostaining

The MEX3C antibodies used were described and validated in our previous study [28]. All the MEX3C antibodies recognize all mouse and human MEX3C variants. In addition, the following antibodies were used: anti-Flag (Sigma, F1804, 1:2000), anti-beta actin (Sigma, A2228, 1:5000), anti-THOC4 (Aly) (Santa Cruz, SC-28729, 1:1000), anti-AP-2 μ2 (AP50) (BD Biosciences, #611351, 1:500), anti-AP-2 β2 (Santa Cruz, sc-6310, 1:1000), anti-AP-2 α (Santa Cruz, 1:1000), anti-HA (Santa Cruz, 1:1000), anti-clathrin heavy chain (Abcam, AB14401, 1:1000), anti-HGS antibody (Abcam, ab56468, 1:1000). These antibodies were validated by the vendors and in multiple publications (see respective vendors’ websites). Horseradish peroxidase (HRP)-conjugated secondary antibodies and chemiluminescent reagents were purchased from ThermoFisher Scientific. The LAS-3000 system (Fujifilm) was used to capture Western blotting images. Densitometry of protein bands was analyzed with Image J software.

### siRNA knockdown experiments

**s**iRNA specific to human *MEX3C* mRNA (5’-CCUAGCAGUUGACUCUCCUGCCUUU-3’) was purchased from Thermo Scientific (CA, RKHD2HSS122041). Control siRNA against firefly luciferase (CUUACGCUGAGUACUUCGA), human *AP2A1* siRNA (AAGAGCAUGUGCACGCUGGCCA), human *AP2M1* siRNA (AAGUGGAUGCCUUUCGGGUCA) [35] were synthesized by Eurofins MWG Operon LLC (Louisville, KY;, all contained a 3’ overhang of dTdT. Human *HGS* siRNA was purchased from GE Dharmacon (Lafayette, CO; ON-TARGETplus HGS siRNA, J-016835-05-0002 and J-016835-06-0002). siRNA transfection of HEK293T cells (final concentration 100 nM in medium) was mediated by Lipofectamine 2000 or Lipodectamine iMAX (ThermoFisher Scientific, Waltham, MA) according to the manufacturer’s instructions. Twelve hours after transfection, medium was changed to serum-free medium or medium containing exosome-free fetal bovine serum (System Biosciences, Palo Alto, CA, EXO-FBS-50A-1) for supernatant collection. When the medium contained exosome-free FBS, the supernatant was collected every 24 hours for 3 days. When serum-free medium was used, the supernatant was collected 3 days after the addition of the serum-free medium. The supernatant were kept at 4°C (for RNase treatment experiments) or -80°C (for all other experiments) before use. After the last supernatant collection, the cells were used for RNA and protein extraction.

### Making doxycycline-inducible MEX3C knockdown HEK293T cell lines

Doxycycline-inducible *MEX3C* shRNA constructs were purchased from GE Dharmacon (Lafayette, CO). Three cell lines were prepared by selecting lentiviral vector infected HEK293T cells with 1 μg/ml puromycin as described previously [28]: a non-targeting control cell line from control clone RHS4743, a cell line from clone V2THS_135328 which targets the coding region of *MEX3C* mRNA (nt 1685–1667 of NM_016626.4), and a cell line from clone V3THS_411913 which targets the 3’ untranslated region of *MEX3C* mRNA (nt 3744–3726). Both *MEX3C* shRNA clones are expected to inhibit transcripts for MEX3C-1, MEX3C-2, and MEX3C-3. The cells were cultured in medium with 1 μg/ml doxycycline for at least 72 hours to induce shRNA expression before any assays.

### EV isolation

Twenty-four hours after siRNA transfection, HEK293T cells were changed to serum-free medium or medium with 10% exosome-free fetal bovine serum. The supernatant was centrifuged at 1000 g 4°C for 30 min to remove cell debris. To isolate EVs, which includes exosomes and microvesicles, 20 ml supernatant was centrifuged at 120,000g (Sorvall SureSpin 630) 4°C for 70 mins. The pellet was washed in an equal volume of ice-cold PBS and again pelleted by centrifuge at 120,000g 4°C for 70 mins. The pellet was then used for miRNA isolation or Western blotting analysis. To separate EVs into exosomes and microvesicles, 20 ml supernatant was first centrifuged at 17,500 g 4°C for 30 mins. The pellet was then washed in an equal volume of PBS and spun down again under the same conditions. This pellet was functionally defined as a microvesicle. The resulted supernatant was then filtered through a 0.22 μm filter to remove particles larger than 200 nm, and centrifuged at 120,000g 4°C for 70 mins to spin down small particles. The pellet was similarly washed with an equal volume of PBS and spun down again. This pellet was functionally defined as an exosome.

### Nanoparticle tracking analysis

Hydrodynamic diameters and concentrations of exosomes and microvesicles were measured using the Nanosight NS500 (Malvern Instruments, UK) running software version NTA3.2. The instrument was primed using phosphate buffered saline, pH 7.4 (PBS) and the temperature was maintained at 25°C. Accurate particle tracking was verified using a 50 nm polystyrene nanoparticle standard (Malvern Instruments) prior to examination of the samples. Purified exosome samples were run undiluted in PBS. Samples containing EVs were diluted 10-fold in PBS. Five measurements (60 sec each) were obtained for each sample. Data are reported as the mean of these measurements ± standard error of the mean.

### RNase treatment of EVs

To test the protection of RNase by membrane, EVs isolated from 20 ml of HEK293T cell conditioned medium cultured in 10% exosome-depleted FBS or serum-free medium were re-suspended in 150 μl PBS and were equally split into three tubes. To one tube 1% NP40 was added and the vesicles were treated on ice for 15 mins to disrupt the membrane structure (NP40 treated). Then 1.25 μl of proteinase K (20 mg/ml) was added to each tube and incubated at 37°C for 30 mins to digest proteins. Proteinase K was then inhibited by addition of PMSF to 5 mM (no heat inactivation was included to avoid possible membrane damage by heating). Finally, 2 μl RNase T1 (Epicentre, 1 Ku/μl) and 6 μl RNase A (Sigma, 1.25 mg/ml) were added to the NP40 treated tube and the test tube, while 8 μl PBS was added to the control tube. All tubes were incubated at room temperature for 15 mins and then at 37°C for 30 mins. The vesicles were then used for miRNA extraction and RT-PCR analysis. It is critical that the conditioned medium and the vesicles have never been frozen after collection for this experiment.

### Cellular and EV RNA isolation

miRNeasy mini kit (Qiagen) was used to isolate total RNA (including miRNA) from cells and EV preparations. For RNA isolation from EVs, 2 μl of *C*. *elegans* miR-39-3p (5 nmol/L) was added to each sample after addition of Qiazol reagent. EV RNA was eluted in 30 μl of DNase- and RNase-free water.

### miRNA analysis

cDNA synthesis was performed with the miScript II RT kit (Qiagen). The HiFlex buffer was used to analyze miRNA, mRNA, and noncoding RNA. For EV samples, 10 ul of RNA from each sample were used for cDNA synthesis regardless of RNA concentration. For gene expression in cellular RNA, *GPADH* and *RPL10* were used as internal controls. For gene expression in EV, *C*. *elegans* miR-39-3p (spiked in during RNA purification) was analyzed. To ensure similar RNA recovery and cDNA conversion during RNA purification and reverse transcription, only samples with close miR-39-3p cycle threshold numbers were used for further analysis.

Real-time PCR was performed on a 7300 Real time PCR system (Thermo Scientific). For human *GAPDH*, *FOS* and *HLA-A2* assays, TaqMan probes (Thermo Scientific) were used. For the remaining genes and miRNAs, a miScript SYBR^®^ Green PCR Kit (Qiagen) was used with gene- specific forward primers (Table B in S1 File) and universal reverse primers provided in the kit. After the PCR amplification, a dissociation program was run and the amplified product was analyzed by electrophoresis to verify the specificity of the amplicon. Relative gene expression levels were calculated using the ΔΔCT method. Each experiment was performed at least twice and triplicated PCR was performed for each sample in real time PCR. Unless otherwise stated, results are presented as mean ± s.e.m.

### Imaging analysis

HEK293 cells on coverslips were transfected with the designated plasmid DNA. After 24 hr, the cells were treated with 0.005% digitonin buffer at room temperature for 3 mins to remove soluble cytoplasmic protein [16]. Then the cells were fixed in 4% paraformaldehyde/PBS, pH7.4 at room temperature for 10 mins, followed by treating with 0.1% Triton X-100/PBS at room temperature for 30 mins. The cells were subsequently stained with respective primary and secondary antibodies for protein subcellular localization. Cells were mounted in mounting medium with or without DAPI, depending on whether there was a blue fluorescence generating tag. The cells were observed under an FV10i confocal microscope (Olympus Corporation, Tokyo, Japan). A 60× oil immersion objective (NA = 1.35) was used to focus the laser and collect the fluorescence. Different fluorescence channels were scanned successively. The laser intensity and sensitivity parameters for each channel were kept constant for all samples in the same experiment.

For co-localization experiments, 5–10 areas of 210 x210 μm were scanned for each experiment. Each scanned area had 10~20 cells positive for both probes. For each area, 8–12 planes were scanned with a step of 1 μm between two planes. Composite images of multiple channels from the same plane were exported. For co-localization analysis, cells from multiple areas and multiple planes from each cell were analyzed. To observe signals in cells with relatively weak expression, cells with saturated signals due to overexpression were excluded from further analysis. The Pearson’s correlation coefficient and the co-localization indices (the percentage of pixels from one channel co-localized with the other channel) were calculated from 10–20 randomly picked cells positive for both labels. Quantitative co-localization analysis was performed with the FluoView FV1000 software.

### Statistical analysis

Two tailed t-tests were performed to compare the means of two groups. To compare the means of three groups, one-way analysis of variance (ANOVA) was performed followed by Tukey’s post-tests. p<0.05 was regarded as statistically significant.

## Results

### MEX3C proteins associate with the adaptor-related protein complex 2 (AP-2)

**Fig 1:**
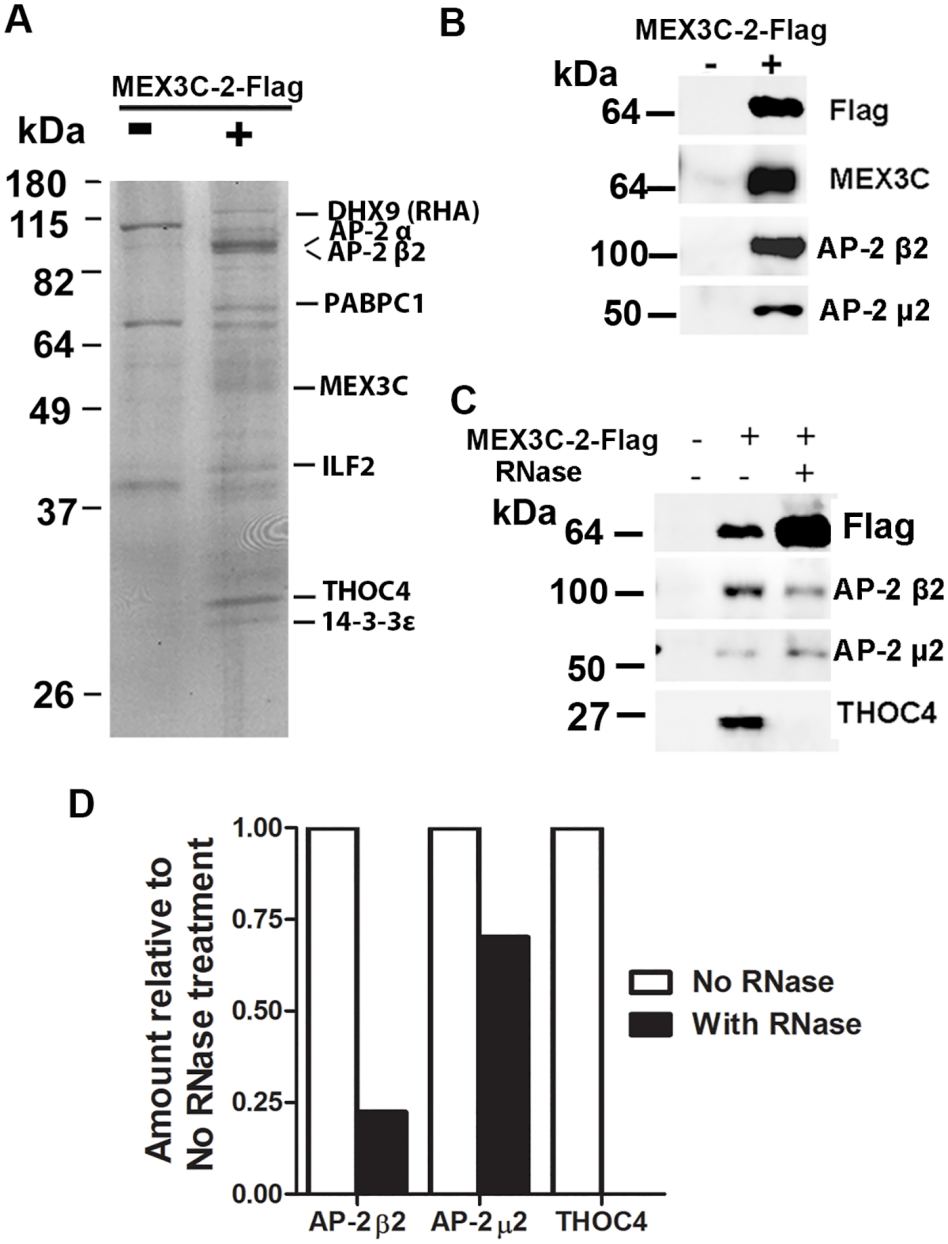
Identification of AP-2 subunits among MEX3C-associated proteins. **A**. Identifying AP-2 α and β2 subunits among MEX3C-associated proteins. MEX3C interacting proteins were pulled down by anti-Flag antibody from cell lysates expressing MEX3C-2-Flag. M: molecular weight marker; kDa: kilodalton. **B**. AP-2 pulldown was MEX3C-dependent. Since affinity purified proteins were loaded, a loading control was unavailable. The lysates were prepared from equal numbers of HEK293 cells transfected with MEX3C-2-Flag expressing DNA or vector DNA, the purification was done in parallel, and equal volumes of eluents from MEX3C-Flag positive (+) and negative (-) lysates were loaded. **C**. RNase treatment did not eliminate MEX3C/AP-2 association. RNase A treatment eliminated MEX3C/THOC4 association, demonstrating that the RNase treatment worked. **D**. Comparisons of relative amounts of protein pulled down by MEX3C-2 with and without RNase treatment. Densitometry of protein bands was analyzed with Image J software. Protein expression was normalized by the respective MEX3C-2-Flag.

To better understand the molecular functions of MEX3C proteins, we searched for MEX3C-associated proteins through immunoprecipitation, SDS-PAGE, and mass spectrometry. Since mRNAs coding for MEX3C-2 is most widely expressed [28], we transiently expressed C-terminal Flag-tagged MEX3C-2 in HEK293 cells, and used anti-Flag antibody-conjugated agarose beads to pull down MEX3C-2 and its associated proteins. After separating the precipitated proteins on SDS-PAGE gels, we reproducibly observed bands from MEX3C-2-Flag positive lysates that were absent from MEX3C-2-Flag negative lysates (Fig 1A). Mass spectrometry analysis identified multiple peptide fragments matching MEX3C-2, confirming the successful recovery of MEX3C-2. Two RNA-binding proteins, PABPC1 and THOC4, were also observed in MEX3C-associated proteins. PABPC1 associates with MEX3C-1 in an RNA-dependent manner [25].

Both the α and β2 subunits of AP-2 complex were observed in the precipitates, with 19 and 23 matching peptides from each subunits (Fig 1A). Cano et al. pulled down the α and β2 subunits of AP-2 complex with MEX3C-1, which shares C-terminal 464 amino acid residues with MEX3C-2 [26]. Due to the established role of AP-2 complex in clathrin-mediated endocytosis and membrane trafficking [11,12], we further examined the MEX3C/AP-2 association and explored whether MEX3C proteins could be involved in membrane-associated RNA sorting.

AP-2 β2 was observed in eluents from MEX3C-2-Flag positive but not MEX3C-2-Flag negative lysates, confirming our mass spectrometry results (Fig 1B). In addition, we also observed the presence of the AP-2 μ2 subunit in MEX3C-2-Flag positive eluents (Fig 1B), consistent with the tight association of the AP-2 subunits. Since MEX3C is an RNA-binding protein, we examined whether the proteins were pulled down merely through RNA bridging. Equal volumes of MEX3C-2-Flag positive cell lysates were treated with or without RNase A before immunoprecipitation. RNase A treatment eliminated the MEX3C/THOC4 association (Fig 1C), demonstrating successful RNase treatment. However, this treatment did not eliminate the MEX3C/AP-2 association, although the quantity of each AP-2 subunits pulled down was reduced after normalization to the mass of MEX3C-2-Flag pulled down (Fig 1C and 1D). RNase treatment reduced the amount of AP-2 μ2 and AP-2 β2 pulled down by MEX3C, suggesting that some AP-2 μ2 and AP-2 β2 might be pulled down by RNA bridging, and/or that RNA may play a role in modulating AP-2 and MEX3C association. Further work is needed to learn the role of RNA in AP-2 and MEX3C interaction. The degree of decrease after RNase treatment seemed to be different between AP-2 μ2 and AP-2 β2. Possible explanations include experimental variation, unequal expression of different AP-2 subunits and different interaction of the AP-2 subunits with MEX3C. More work is needed to determine whether RNase treatment affects the amount of AP-2 μ2 and AP-2 β2 pulled down differently. Nevertheless, our data suggest that the MEX3C/AP-2 association could not be explained only by RNA bridging.

### Sequences around the KH domain shared by all MEX3C variants are required for MEX3C/AP-2 association

**Fig 2:**
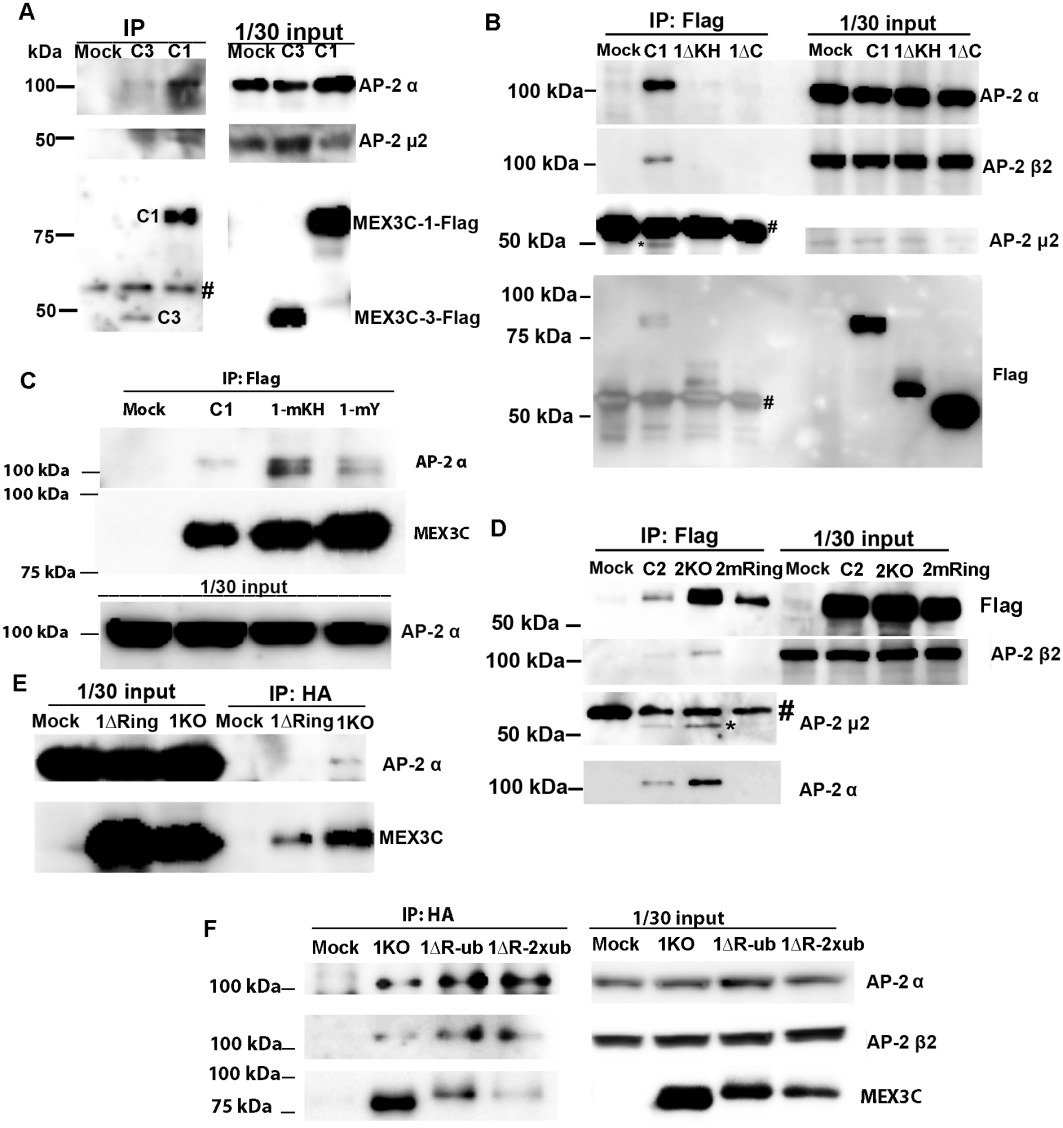
Mapping the MEX3C regions mediating MEX3C/AP-2 interaction. **A**. MEX3C-1 and MEX3C-3 pull down endogenous AP-2 subunits. Transiently expressed MEX3C-1 and MEX3C-3 were Flag-tagged. C1 and C3 indicate MEX3C-1-Flag and MEX3C-3-Flag respectively. # indicates the IgG band. **B**. Deleting the central region or the C-terminal 249AA of MEX3C-1 abolished MEX3C/AP-2 interaction. MEX3C-1 (C1 in Figure), MEX3C-1-ΔKH (1ΔKH) and MEX3C-1-ΔC (1ΔC) were Flag-tagged and detected with anti-Flag antibody. MEX3C-1-ΔC appeared at the same position as the IgG heavy chain. # and * indicate the IgG and the AP-2 μ2 bands, respectively. **C**. Mutating the YXXΦ-like motifs (lane 1-mY) or the amino acids necessary for RNA binding (lane 1-mKH) did not affect MEX3C/AP-2 interaction. Transiently expressed MEX3C-1, MEX3C-1-mY, and MEX3C-1-mKH were Flag-tagged and immunoprecipitated by anti-Flag antibody to detect co-immunoprecipitated AP-2 subunits. The AP-2 α subunit pulled down by both mutants contained a smaller band in addition to the normally observed large band. **D**. The ring finger domain of MEX3C is necessary for MEX3C/AP-2 interaction. MEX3C-2 (C2 in Figure), MEX3C-2-KO (all lysine residues were mutated to arginine, lane “2KO”), and MEX3C-2-mRing (the C3HC4 motif in the MEX3C-2 ring finger domain was mutated to A3NC4, lane “2mRing”) were Flag-tagged. # indicates the IgG band and * indicates the AP-2 μ2 band. **E**. Deleting the ring finger domain (C-terminal 53 AA) of MEX3C-1 abolished MEX3C/AP-2 interaction. 1ΔRing: MEX3C-1-KO-ΔRing, all lysine residues of MEX3C-1 were mutated to arginine and the ring finger domain was deleted. 1KO: All lysine residues in MEX3C-1 were changed to arginine. Both mutants were HA-tagged and immunoprecipitated by anti-HA antibody to detect AP-2 subunits. **F**. Addition of ubiquitin chain(s) to MEX3C-1-KO-ΔRing restored MEX3C/AP-2 interaction. 1ΔR-ub: MEX3C-1-KO-ΔRing-UbKO, MEX3C-1-KO-ΔRing fused to one ubiquitin chain whose lysine residues were all mutated to arginine; 1ΔR-2xub: MEX3C-1-KO-ΔRing-2xUbKO, MEX3C-1-KO-ΔRing fused to two ubiquitin chains whose lysine residues were all mutated to arginine. All three mutants were HA-tagged. For **A**~**F**, “Mock” indicates vector-DNA transfected cells.

**Fig 3:**
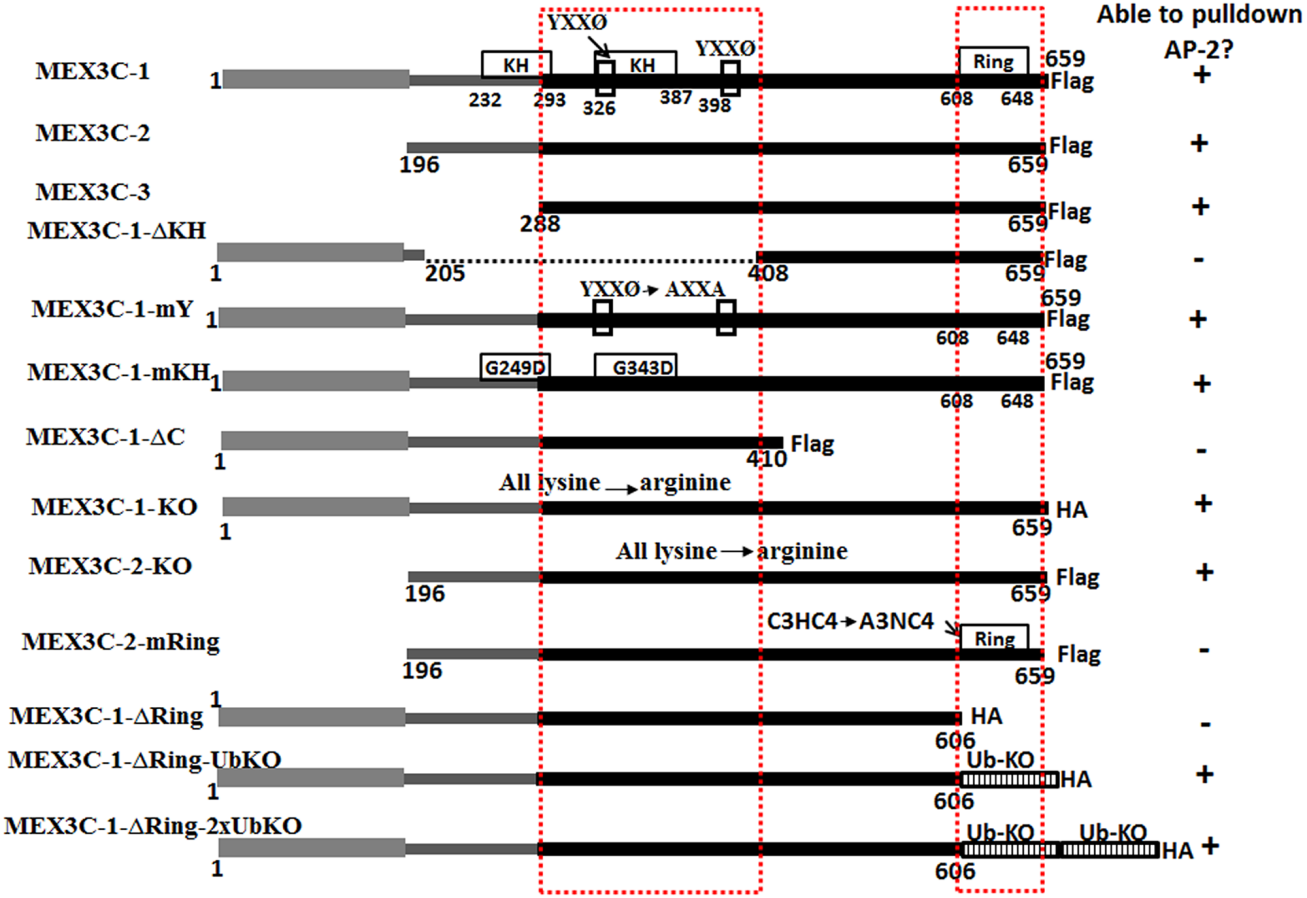
Summary of interaction data of MEX3C variants and mutants with AP-2. Black boxes indicate sequences that were identical among MEX3C-1, MEX3C-2, and MEX3C-3. Thin gray boxes indicate sequences shared by MEX3C-1 and MEX3C-2. Thick gray boxes indicate sequences unique to MEX3C-1. Unfilled boxes indicate motifs and domains studied. Boxes filled with vertical lines indicate ubiquitin chains with lysine changed to arginine. Dashed lines indicate deleted regions in deletion mutants. Dashed red boxes indicate regions essential for MEX3C/AP-2 interaction. Sequence numbering is based on human MEX3C-1. “+” indicates able to pull down AP-2 complex in immunoprecipitation experiments while “-” indicates the inability to do so. The tag (Flag or HA) for each variant and mutant is indicated. Detailed immunoprecipitation data are presented in Fig 2.

Consistent with the factor that MEX3C-1 contains all the sequences of MEX3C-2, MEX3C-1 could also pull down the α and μ2 subunits of AP-2 complex (Fig 2A). MEX3C-3 only contains the C-terminal 372 AA of MEX3C-1 and MEX3C-2 (see Fig 3) and could also pull down AP-2 subunits (Fig 2A). The data suggest that the sequences common to the three MEX3C variants are sufficient to mediate MEX3C/AP-2 interaction.

To further map the regions in MEX3C proteins important for interacting with the AP-2 complex, we tested the interaction of AP-2 with various MEX3C deletion and point mutants. MEX3C-3 has only one KH domain (the second KH domain of MEX3C-1 and 2) and can pull down the AP-2 complex. To determine whether sequences of this KH domain are necessary for MEX3C/AP-2 interaction, we deleted both KH domains in MEX3C-1 (AA 206–407 of MEX3C-1) and found that it could no longer pull down AP-2 subunits (Fig 2B, lane 1ΔKH). The sequences between AA 288 and 407 were necessary for MEX3C-1/AP-2 interaction (see Fig 3 for a summary of all interaction data).

Since ^335^YRVV and ^398^YIEL in this region resemble the tyrosine-based sorting motif YXXΦ (X indicates any amino acid and Φ a bulky hydrophobic amino acid) through which membrane-associated cargo proteins associate with the AP-2 complex [18,19], we asked whether the YXXΦ-like motifs in MEX3C are important for MEX3C/AP-2 association. We mutated the “Y” and “Φ” residues important for cargo/AP-2 association to alanine, thus changing the two YXXΦ-like motifs to AXXA, and found that this alteration did not affect MEX3C/AP-2 interaction (Fig 2C, lane 1-mY). The data suggest that unlike in membrane proteins, YXXΦ in MEX3C is not responsible for its AP-2 association. To check whether the RNA-binding activity of the KH domain is necessary for MEX3C/AP-2 association, we mutated ^249^Gly and ^343^Gly to Asp, which abolishes MEX3C’s RNA-binding activity [25,26]; it also did not abolish MEX3C/AP-2 interaction (Fig 2C, lane 1-mKH). Thus, sequences spanning the second KH domain are important for MEX3C/AP-2 interactions, but RNA-binding activity and the YXXΦ-like motifs are irrelevant.

### The E3-ligase activity of the MEX3C ring finger domain is also important for MEX3C/AP-2 association

To explore whether the C-terminal part of MEX3C proteins plays a role in the MEX3C/AP-2 association, we deleted the C-terminal 248 AA (AA 411–659) of MEX3C-1 and examined the interaction with AP-2. This C-terminal truncated mutant did not associate with AP-2 (Fig 2B, lane 1ΔC). Because this region contains a ring finger domain with ubiquitin E3 ligase activity, we examined whether the ring finger domain is involved in MEX3C/AP-2 association. Although wild-type MEX3C-2 could associate with AP-2 (Fig 2D, lane C2), mutating MEX3C-2’s C3HC4 motif in the ring finger domain (essential for ubiquitin E3 ligase activity) to A3NC4 (single-letter amino acid code) abolished the MEX3C/AP-2 interaction (Fig 2D, lane 2mRing), suggesting that the ring finger domain of the C-terminus is important for MEX3C/AP-2 interaction.

The ubiquitin E3-ligase activity of the MEX3C ring finger domain can ubiquitinate MEX3C itself [26]. To test whether MEX3C ubiquitination is important for the MEX3C/AP-2 interaction, we changed all lysine residues of MEX3C-2 to arginine, eliminating its ability to be ubiquitinated. This MEX3C-2 mutant could still interact with AP-2 (Fig 2D, lane 2KO), suggesting that ubiquitination of MEX3C itself is not necessary for MEX3C/AP-2 interactions.

Nonetheless, it is possible that MEX3C proteins ubiquitinate unidentified MEX3C-interacting proteins, which subsequently facilitate the MEX3C/AP-2 interaction. To test whether ubiquitin modification is involved in MEX3C/AP-2 interaction, we made three mutants and fusion proteins: 1) MEX3C-1-KO-ΔRing, with all lysine residues of MEX3C-1 mutated to arginine, and the ring finger domain encoded by the C-terminal 53 AA deleted; 2) MEX3C-1-KO-ΔRing-UbKO, where MEX3C-1-KO-ΔRing was fused to one ubiquitin chain whose lysine residues were all mutated to arginine; and 3) MEX3C-1-KO-ΔRing-2xUbKO, where MEX3C-1-KO-ΔRing was fused to two ubiquitin chains whose lysine residues were all mutated to arginine. Absence of lysine residues in the mutants (including the HA-tag) ensured that the mutant proteins could not be further ubiquitinated. Consistent with our earlier observation, MEX3C-1-KO-ΔRing did not interact with AP-2 (Fig 2e, lane 1ΔRing), although MEX3C-1-KO did (Fig 2E, lane 1KO). However, addition of one ubiquitin chain or two tandem ubiquitin chains (all K in the ubiquitin changed to R), to the C-terminus of MEX3C-1-KO-ΔRing restored MEX3C/AP-2 interaction (Fig 2F, lanes 1ΔR-ub and 1ΔR-2xub, see Fig 3 for sequence composition of the mutants).

With increased numbers of ubiquitin chains, expression of the MEX3C fusion proteins decreased, while the ability to pull down AP-2 complex increased (Fig 2F, lanes 1ΔR-ub and 1ΔR-2xub). Quantitative RT-PCR detected comparable *MEX3C* mRNA levels in cells transfected with these mutants (all had about 1,000 times more *MEX3C* mRNA than cells without transfection), suggesting that post-transcriptional mechanisms were responsible for these results. Although the sequences of the mutants were confirmed by DNA sequencing, MEX3C-1-KO-ΔRing-UbKO and MEX3C-1-KO-ΔRing-2xUbKO did not show overt differences in size on SDS-PAGE gels.

We did not perform *in vitro* pulldown assays to determine whether the MEX3C and AP-2 interaction is direct or indirect, nor did we determine which subunits of AP-2 are involved in MEX3C/AP-2 interaction. Nevertheless, our data show that although ubquitination of MEX3C itself was not necessary for MEX3C/AP-2 interaction, the ring finger ubiquitin E3 ligase activity of MEX3C was. In summary, two regions in MEX3C proteins are important for the MEX3C/AP-2 interactions: sequences in the KH domain shared by MEX3C-1, MEX3C-2 and MEX3C-3, and the ring finger domain at the C-termini of all MEX3C variants (Fig 3).

### MEX3C proteins are recruited to the endolysosomal compartment

**Fig 4:**
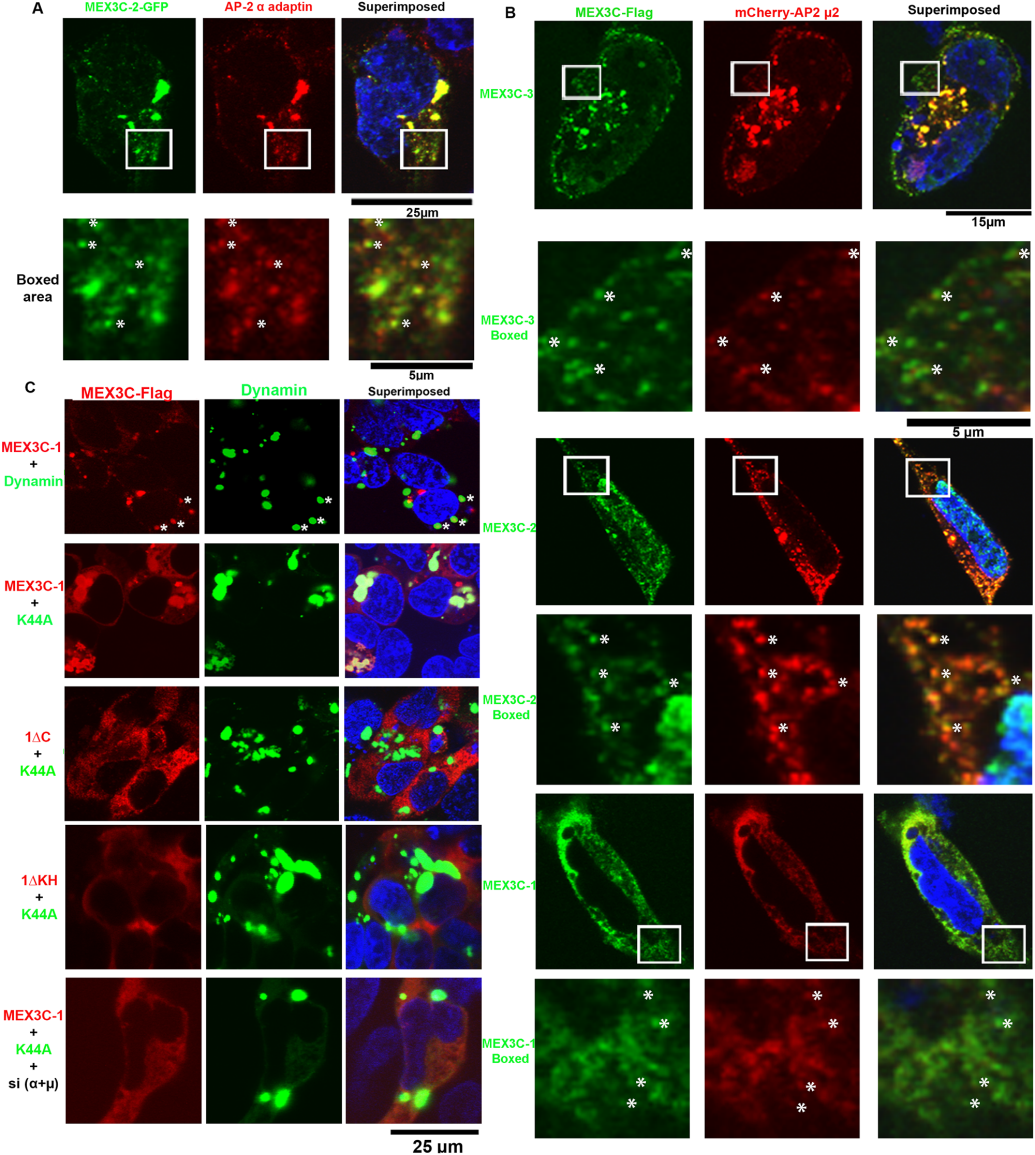
Subcellular localization of MEX3C in the endolysosome compartment. **A**. Co-localization of MEX3C-2 (GFP-tagged) with an endogenous AP-2 α subunit. Endogenous AP-2 α was immunostained by specific antibody. The boxed area was shown at high magnification in the following row. Co-localized signals were marked by *. **B**. Co-localization of transiently expressed MEX3C variants (Flag-tagged, stained by anti-Flag antibody) with transiently expressed AP-2 μ2 (mCherry-tagged). The boxed area was shown at high magnification in the following row. Co-localized signals are marked by *. **C**. GTP binding defective dynamin^K44A^ mutant trapped wild-type MEX3C-1, but not MEX3C-1 mutants unable to interact with AP-2, at dynamin^K44A^ enriched foci. K44A: Dynamin^K44A^ mutant; 1ΔC: MEX3C-1ΔC, MEX3C-1 C-terminal 247AA deleted; 1ΔKH: MEX3C-1ΔKH, MEX3C-1 KH domains (AA 206–407) deleted. Shown are representative images of multiple double-positive cells. Quantitative data are presented in Fig A in S1 File.

Next, we examined the subcellular localization of MEX3C and AP-2. Since our MEX3C antibodies did not detect endogenous MEX3C proteins in immunostaining assays, we examined the co-localization of transiently expressed MEX3C proteins with AP-2 complex. MEX3C-2-GFP showed co-localization with endogenous AP-2 α subunit (Fig 4A). In addition, transiently expressed, Flag-tagged MEX3C-1, MEX3C-2 and MEX3C-3 all co-localized with a transiently expressed mcherry-tagged AP-2 μ2 subunit in the cytoplasm, but not in the nucleus (Fig 4B). The co-localization of MEX3C proteins with the AP-2 complex in the cytoplasm is consistent with our biochemical data that these proteins interact with the AP-2 complex.

The primary function of AP-2 complex is facilitating the formation of clathrin-coated vesicles at the cell membrane [11,12]. The MEX3C/AP-2 association prompted us to examine whether MEX3C proteins could associate with these clathrin-coated vesicles. Due to the highly dynamic nature of membrane trafficking, we studied the localization of MEX3C proteins when endocytosis was blocked by a dynamin^K44A^ mutant (GFP-tagged) defective in GTP binding and could not pinch the forming vesicles [36]. When wild-type dynamin (GFP-tagged) was co-expressed with MEX3C-1 (mCherry-tagged), only a small fraction of dynamin-positive foci were also positive for MEX3C-1 (Fig 4C, first row, marked by *). However, when dynamin^K44A^ mutant was co-expressed with MEX3C-1, all dynamin^K44A^-positive foci were enriched with MEX3C-1 (Fig 4C, second row), suggesting that MEX3C-1 was trapped at the location of clathrin-coated vesicle formation. Similarly, transiently expressed MEX3C-2 and MEX3C-3 were also enriched in dynamin^K44A^-positive foci (Fig A in S1 File). When MEX3C-1ΔC and MEX3C-1ΔKH (both unable to interact with AP-2) were co-expressed with dynamin^K44A^, dynamin^K44A^-positive foci were no longer enriched with these mutants (Fig 4C, third and fourth row). Since both MEX3C-1ΔC and MEX3C-1ΔKH could not interact with AP-2, it suggests that the ability to interact with AP-2 is necessary for MEX3C to be enriched at the dynamin^K44A^ foci. Supporting our observations, when AP-2 α and μ subunit expression was inhibited by validated siRNAs, the degree of MEX3C-1 enrichment at the dynamin^K44A^ foci was greatly attenuated (Fig 4C, last row). The Pearson’s correlation coefficient of MEX3C-1 and dynamin^K44A^ was significantly larger than those of MEX3C-1 and wild type dynamin, MEX3C-1ΔC and dynamin^K44A^, and MEX3C-1ΔKH and dynamin^K44A^ (p<0.0001, n = 10 to 20, Fig A in S1 File, Panel b). Inhibiting AP-2 subunits significantly decreased the percentage of dynamin^K44A^ pixels co-localized with MEX3C-1 (p<0.0001, n = 10 to 20, Fig A in S1 File, Panel c). The data suggest that MEX3C proteins are most likely recruited to clathrin-coated vesicles during endocytosis through interacting with AP-2.

**Fig 5:**
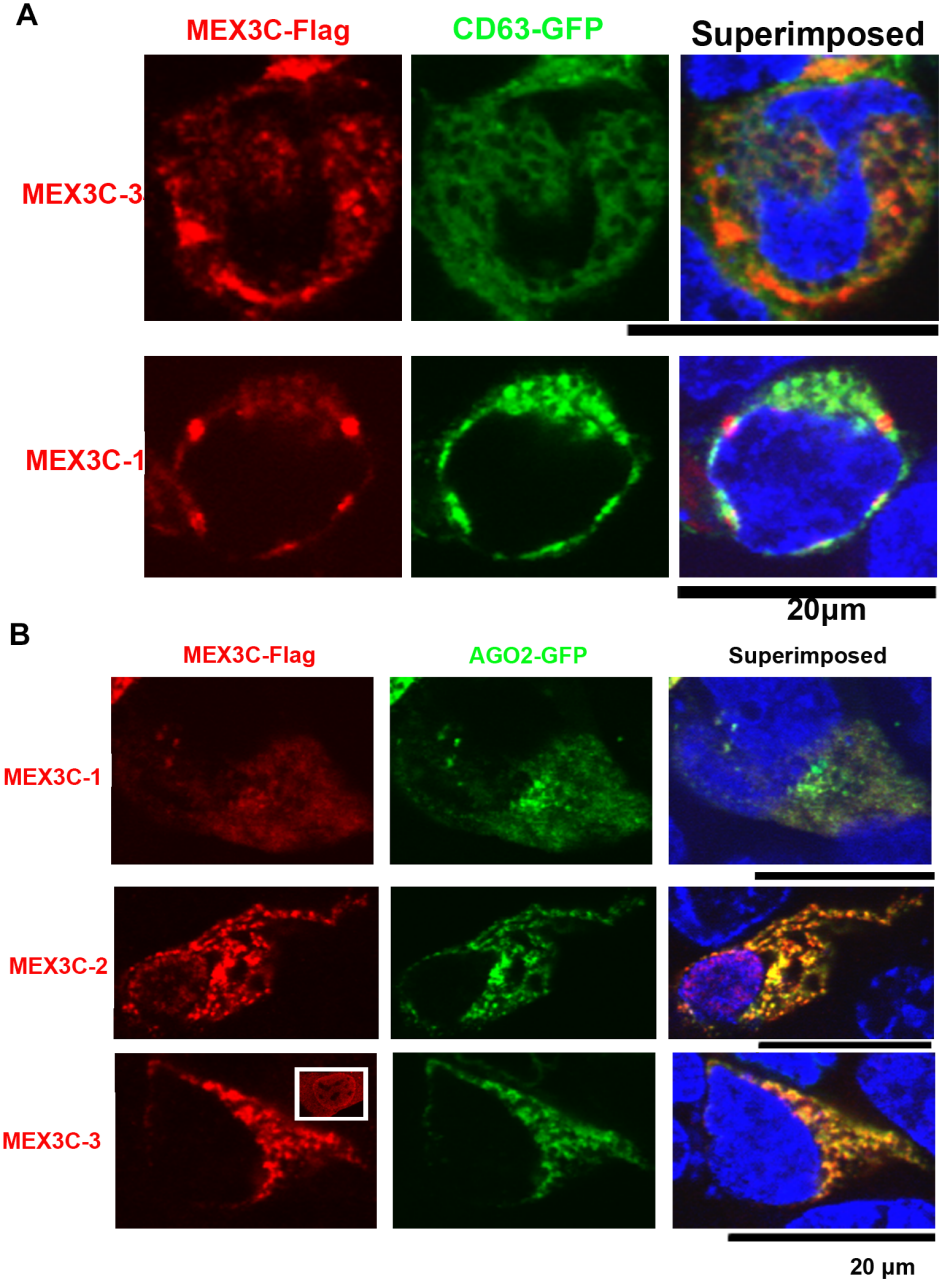
Co-localization of MEX3C proteins with CD63 and AGO2. **A**. MEX3C proteins showed partial co-localization with CD63. **B**. MEX3C proteins showed a high degree of co-localization with AGO2. The boxed image shows the ubiquitous localization of singly expressed MEX3C-3. In MEX3C-3 and AGO2 double-positive cells, all MEX3C-3 protein was cytoplasmic. For **A**-**B**, MEX3C proteins were Flag-tagged; CD63 and AGO2 were GFP-tagged and their similarity to endogenous proteins was validated by respective donating investigators (see Acknowledgments for list of investigators). Shown are representative images of multiple double-positive cells. Nuclei (stained by DAPI) were pseudocolored blue.

To further examine associations between MEX3C with the endolysosomal compartment, we examined the co-localization of MEX3C proteins with early endosomal protein RAB5A, recycling endosomal protein RAB11, multivesicular body (MVB) marker protein CD63, and lysosomal protein LAMP1. MEX3C proteins showed little co-localization with RAB5A, RAB11, LAMP1, or ER marker (Fig B in S1 File). However, both MEX3C-3 and MEX3C-1 (Flag-tagged) showed partial co-localization with CD63 (GFP-tagged) in the cytoplasm (Fig 5A). Although 50% of MEX3C-2 singly positive cells had cytoplasmic MEX3C-2, all MEX3C-2 and CD63 double-positive cells had only nuclear MEX3C-2, preventing us from examining their co-localization. MEX3C-1 degradation was significantly inhibited by the lysosome acidification inhibitor Bafilomycin A1 (Fig C in S1 File), suggesting the involvement of the lysosome in MEX3C-1 degradation. This observation is consistent with MEX3C’s association with the endolysosomal system.

Argonaute 2 (AGO2) interacts with MEX3C-1 [25] and associates with multivesicular bodies [37,38]. Indeed, transiently expressed MEX3C-1, MEX3C-2, and MEX3C-3 (all Flag-tagged) showed high degree of co-localization with AGO2 (GFP-tagged) (Fig 5B). MEX3C-2 localizes in the nucleus and the cytoplasm; however, co-localization was only observed in the cytoplasm (Fig 5B, second row). Singly expressed MEX3C-3 was observed in the nucleus of all positive cells (insert in third row of Fig 5B). However, co-expression of AGO2 with MEX3C-3 removed nuclear but not cytoplasmic MEX3C-3, and the cytoplasmic MEX3C-3 showed a high degree of co-localization with AGO2 (Fig 5B, third row). The MEX3C-AGO2 co-localization data are consistent with previous observations of MEX3C-AGO2 interaction [25], and further support the association of MEX3C proteins with the endolysosomal system. Although GW182 interacts with AGO2 and is essential for miRNA mediated repression [39], MEX3C-1 did not show evident co-localization with GW182 (Fig D in S1 File). It is likely that the subset of AGO2 interacting with MEX3C proteins are not the same as those interacting with GW182.

### MEX3C and AP-2 are involved in extracellular but not cellular miR-451a expression

Exosomes are generated from the fusion of multivesicular bodies with the cell membrane [40,41] and some miRNAs are preferentially sorted to exosomes [1,2] through a mechanism that remains unclear. The association of RNA-binding E3 ligase MEX3C with the endolysosome system prompted us to examine whether MEX3C could be involved in miRNA exosomal sorting.

**Fig 6:**
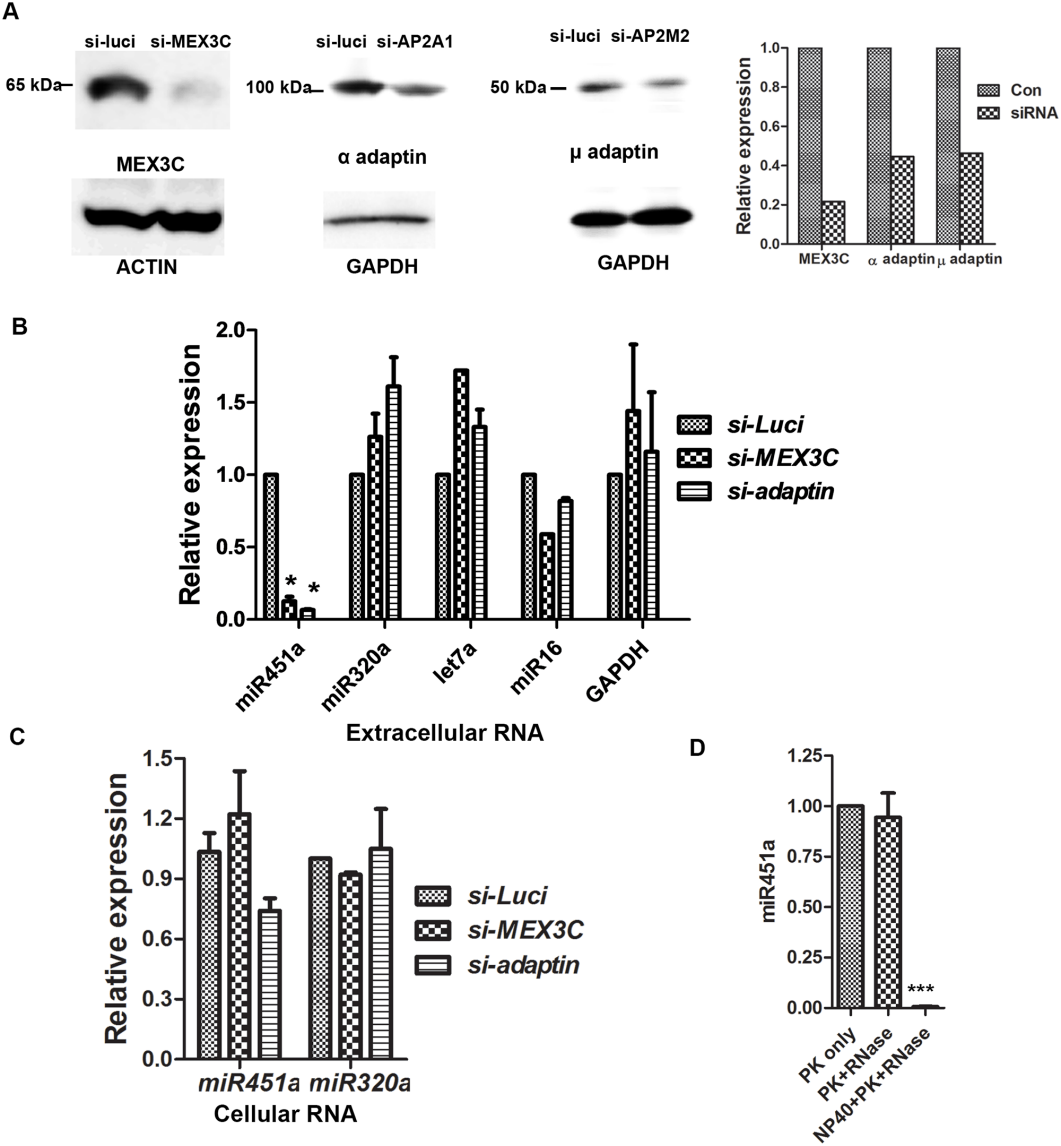
MEX3C and AP-2 are involved in EV miR-451a secretion. **A**. Western blotting confirmed efficiency of the MEX3C and AP-2 siRNAs. MEX3C was detected 24 hours after siRNA transfection; the loading control was β-actin. AP-2 α and AP-2 μ2 were examined 84 hours after siRNA transfection (after EV collection). The loading control was GAPDH. The panel on the right shows the relative expression after normalized to loading control determined by densitometry. Results are representative of two independent experiments. **B**. Real-time PCR analysis of EV RNA expression after MEX3C or AP-2 inhibition. Because inhibiting AP-2 α or AP-2 μ, or AP-2 α and AP-2 μ in HEK293T cells showed similar effects, these data were combined. * indicates p<0.001 by Tukey's multiple comparison test following ANOVA. For miR-451a and miR-320a, N = 3; for the others, N = 2. **C**. MEX3C or AP-2 inhibition did not affect cellular miR-451a expression (N = 3). **D**. EV miR-451a was protected from RNase by membrane (N = 5). ***, p<0.0001 between protease K and RNase treated samples with and without NP40 pre-treatment, analyzed by Tukey's multiple comparison test following ANOVA. For **B**, **C** and **D**, means ± standard error (s.e.m.) are shown.

We used siRNAs specific to *MEX3C* (to inhibit MEX3C), *AP2A1/AP2A2* (to inhibit AP-2 α), and *AP2M1* (to inhibit AP-2 μ) to examine the role of AP-2 complex and MEX3C in sorting miRNA into the EV in HEK293T cells. The siRNAs for AP-2 subunits were validated in previous studies [35]. We confirmed that all the siRNAs could efficiently inhibit expression of the respective genes (Fig 6A). We reasoned that examining miRNA species enriched in EVs would increase the chance of finding miRNA species affected by MEX3C or AP-2 inhibition. In previous studies, miR-451a, miR-146a-5p, miR-150-5p, miR-320a, and let-7a-5p preferentially associated with EVs [2,42]. These miRNAs were examined to see whether MEX3C or AP-2 inhibition affects their EV expression. miR-16-5p has a very low EV to intracellular ratio, but is abundant in EVs due to its high total expression [2]. Two mRNA species, *GAPDH* and *RPL0*, were compared as controls. miR-451a in EVs was clearly decreased by MEX3C or AP-2 knockdown (Fig 6B). Except for miR-146a-5p and miR-150-5p (which had insufficient EV expression to be conclusive), none of the other RNAs examined was affected by MEX3C or AP-2 inhibition. Inhibiting expression of AP-2 α, or AP-2 μ, or AP-2 α and AP-2 μ, all decreased expression of miR-451a, but not miR-320a, miR-16-5p or *GAPDH*, in HEK293T EVs (Fig 6B). We examined the effects of MEX3C or AP-2 inhibition on EV secretion, and found that MEX3C or AP-2 inhibition did not decrease EV secretion (Table C in S1 File). This observation is also consistent with our finding that no RNAs other than miR451a were decreased in EVs when MEX3C or AP-2 was inhibited.

MEX3C’s interaction partner AGO2 [25] is necessary for miR-451a biogenesis [43–45]. However, cellular miR-451a expression was not affected by MEX3C or AP-2 inhibition (Fig 6C). Without knowing the percentage of total miR-451a that is secreted to EVs, it is difficult to determine whether the total miR-451a generated is affected. However, if MEX3C and AP-2 are involved in miR-451a biogenesis, cellular miR-451a should be similarly decreased upon MEX3C or AP-2 inhibition. Thus our data argue against the possibility that EV miR-451a decrease after MEX3C or AP-2 inhibition is a result of decreased cellular miR-451a biogenesis.

It is uncertain whether MEX3C or AP-2 inhibition affects total EV RNA due to the difficulty in accurately quantifying the small amount of EV RNA isolated from the supernatant. However, indirect evidence suggests that MEX3C or AP-2 inhibition may not affect total EV RNA secretion, since only miR-451a was affected by MEX3C or AP-2 knockdown. However, miR-451a was not the most abundant RNA in the EVs. For the same EV RNA sample, the cycle threshold number for miR-451a is always 5 cycles more than that of miR-16 (21 versus 16), and reductions in EV miR-451a would have little effect on the amount of total EV RNA. Thus, our data suggest that MEX3C or AP-2 knockdown affects the EV secretion of miR-451a, but not the total EV miRNA.

To test whether miR-451a is outside or inside the membrane structures, we treated the EVs (never frozen) with proteinase K followed by RNase A and RNase T1 digestion. This treatment did not change vesicle-associated miR-451a. However, treating the vesicles with 1% NP40 before proteinase K and RNase treatment degraded 99% of the vesicle-associated miRNA (Fig 6D). Thus, miR-451a was inside the membranous structures.

### MEX3C and AP-2 are involved in miR-451a exosome secretion

**Fig 7:**
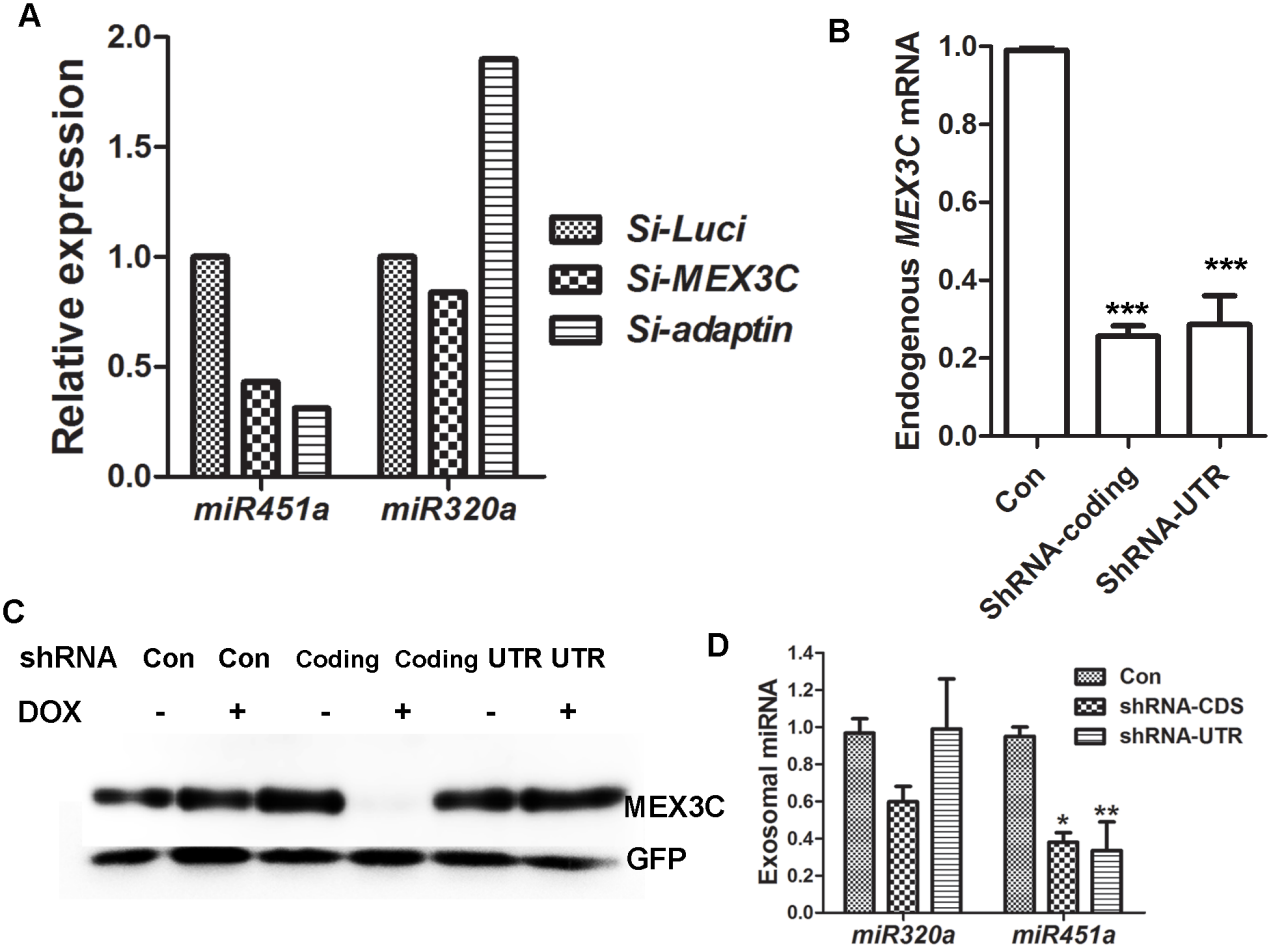
MEX3C and AP-2 are involved in exosomal miR-451a secretion. **A**. siRNA-mediated MEX3C or AP-2 inhibition reduced miR-451a in exosome-enriched preparations. For AP-2 inhibition, siRNAs for *AP2A1* and *AP2M1* were transfected simultaneously. Shown are representative data from two independent transfections. **B**. DOX-induction inhibited *MEX3C* expression at the RNA level (N = 3). **C**. shRNA targeting the *MEX3C* coding region inhibited MEX3C expression from pFlag-MEX3C-1 after DOX-induction (N = 2). Co-transfected EGFP expression was used as the control for transfection efficiency and loading. **D**. DOX-induced MEX3C inhibition decreased exosomal miR-451a but not miR-320a expression (N = 3). For **B** and **D**, the mean ± s.e.m. are presented. *, ** and *** indicate p<0.05, 0.01 and 0.0001 when compared with control in Tukey’s multiple tests (**B**) and Bonferroni posttests (**D**).

Exosomes are the EVs generated from multivesicular bodies. The association of MEX3C with AP-2 and the endolysosome system prompted us to examine whether they are involved in exosomal miR-451a secretion. To enrich exosomes, we first removed dense particles by centrifugation at 17500g for 30 mins, then removed large particles by filtering through a 0.22 μm filter. The resulted supernatant was centrifuged to pellet the exosomes-enriched vesicles. Inhibiting MEX3C or AP-2 decreased miR-451a in exosome-enriched preparations (Fig 7A), although to a lesser extent than in EVs. We reasoned that this could be caused by the heterogeneity of the exosomes. Spinning at 17500g for 30 mins and filtering through a 0.22 μm filter might have removed some exosomes containing miR-451a. Accordingly, exosome-enriched vesicles contained over 60 fold more miR-451a (67.91±11.44, n = 5) than microvesicles (the EVs pelleted after spinning at 17500g for 30 mins) prepared from the same supernatant.

Inhibiting expression of AP-2 α, AP-2 μ, or AP-2 α and AP-2 μ showed similar effects on miR-451a expression in EVs. To address the unlikely possibility of off-target effects of *MEX3C* siRNA, we further examined the effects of *MEX3C* knockdown on exosomal miR-451a secretion in HEK293T cell lines expressing doxycycline (dox)-inducible *MEX3C* shRNAs. HEK293T cell lines were made to express a non-targeting shRNA (control), a shRNA targeting *MEX3C* coding region (V2THS_135328, named shRNA-coding) and a shRNA targeting *MEX3C* 3’-untranslated region (V2THS_411913, named shRNA-UTR). Both *MEX3C* shRNAs target the *MEX3C* mRNA sequence different from that of the *MEX3C* siRNA used in the previous experiments. Using real-time RT-PCR, we found that after DOX addition cells expressing shRNA coding and shRNA-UTR both reduced *MEX3C* mRNA to 30% of that of control cells (Fig 7B).

Considering the difficulty of detecting endogenous MEX3C protein, we examined the inhibitory effects of the shRNAs on transiently expressed MEX3C. The cells were transfected with the plasmid DNA expressing MEX3C-1-Flag, which contained the *MEX3C-1* mRNA coding sequence but not the 3’ UTR. As expected, MEX3C-1-Flag was substantially inhibited in DOX-treated cells expressing shRNA-coding, but not in DOX-treated cells expressing shRNA-UTR or the control shRNA (Fig 7C). We then cultured the cells in serum-free, DOX-containing medium for 48 hours and isolated exosomal miRNA from the culture medium. Using real-time RT-PCR, we found that exosomal miR-451a but not miR-320a was significantly decreased in cells expressing *MEX3C* shRNA (Fig 7D). In these experiments, serum-free medium was used to exclude possible interference from residual exosomes of exosome-depleted FBS. In addition, *MEX3C* inhibition was achieved by DOX induction rather than siRNA transfection. Again, DOX induced *MEX3C* shRNA expression did not decrease the total exosome secretion (Table D in S1 File). Since three different *MEX3C* siRNA and shRNA produced similar effects on miR-451a expression in EVs, we could rule out the possibility of siRNA off targets.

### MEX3C does not directly bind miR-451a

To test whether MEX3C specifically binds to miR-451a, we immunoprecipitated MEX3C-1 or MEX3C-1-mKH with an anti-Flag antibody from HEK293T cells transfected with empty vector DNA, and plasmid DNA expressing MEX3C-1 and MEX3C-1-mKH (MEX3C-1 and MEX3C-1-mKH were Flag-tagged). Since MEX3C-1-mKH contains mutations that disrupt RNA-binding activity [25,26], we expected it to lose the ability to bind its cognate RNA targets but not indirectly associated RNAs. Thus, only those RNAs enriched by MEX3C-1 but not by MEX3C-1-mKH would be MEX3C’s direct RNA targets.

We compared the amounts of *FOS*, miR-320a and miR-451a immunoprecpitated by anti-Flag antibody. The anti-Flag antibody pulled down similar amounts of MEX3C-1 and MEX3C-1-mKH proteins (Fig E in S1 File, left). However, MEX3C-1 pulled down 22 times more *FOS* mRNA than MEX3C-1-mKH, consistent with our recent observation that MEX3C-1 functioned as an adaptor for exportin 1-mediated *FOS* mRNA nuclear export [28]. Nevertheless, neither MEX3C-1 nor MEX3C-1-mKH enriched miR-451a or miR-320a. In addition, the amount of miR-451a pulled down by MEX3C-1 was not different than that pulled down by MEX3C-1-mKH(Fig E in S1 File, right). Thus, our data suggest that miR-451a is not a direct MEX3C target.

### miR-451a exosomal secretion was impaired by inhibiting ceramide but not the ESCRT-0 component HGS

**Fig 8:**
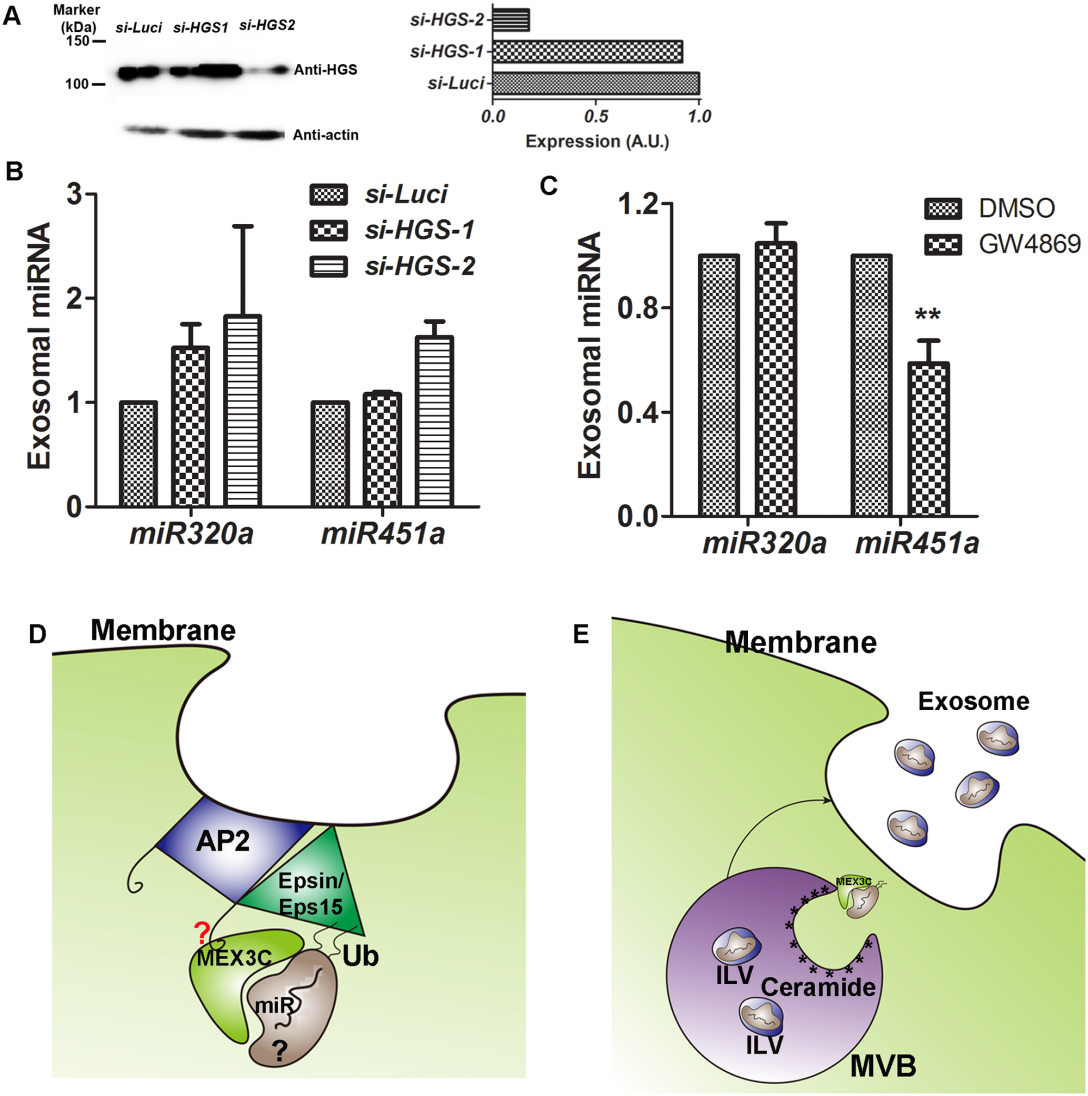
Role of AP-2 and MEX3C in exosomal miR-451a secretion. **A**. HGS was efficiently inhibited by siRNA *si-HGS-2* but not *si-HGS-1*. Right: Expression as measured by densitometry (normalized with β-actin expression). Shown are representative results from two experiments. **B**. Inhibiting HGS expression failed to decrease exosomal miR-451a expression (N = 2). **C**. GW4869 significantly inhibited exosomal miR-451a expression (N = 3). The mean ± s.e.m. are shown. * indicates p<0.05 compared with DMSO control in Bonferroni posttests following ANOVA. **D**. Proposed model for AP-2/MEX3C interaction. At least two regions in MEX3C are necessary for its interaction with AP-2 complex. The red question mark indicates uncertainty whether the MEX3C/AP-2 interaction is direct or indirect; the black question mark indicates that the identities of the substrates ubiquitinated by MEX3C ring finger domain, and the proteins that directly bind microRNA, are unknown. **E**. Proposed role of MEX3C in ceramide-mediated miRNA exosomal sorting. MEX3C increases the association of microRNA to the endosome. ILV: intraluminal vesicles; MVB: multivesicular body; *: ceramide. It is unknown whether MEX3C itself is sorted into the ILVs, so MEX3C was not shown in ILVs.

The ESCRT-0 component HGS (hepatocyte growth factor-regulated tyrosine kinase substrate, also known as HRS) recognizes ubiquitinated cargos of multivesicular bodies and facilitates their sorting into intraluminal vesicles. MEX3C’s association with endosomes and its ubiquitin E3 ligase activity prompted us to ask whether MEX3C could play a role in miR-451a sorting in an ESCRT-dependent manner. We inhibited HGS expression through siRNA interference. Two siRNAs from GE Dharmacon were tested: J-016835-05-0002 (*si-HGS-1*) and J-016835-06-0002 (*si-HGS-2*). *si-HGS-2* but not *si-HGS-1* efficiently inhibited HGS expression (Fig 8A). However, *si-HGS-2* transfection failed to decrease miR-451a exosomal expression (Fig 8B), suggesting that HGS, and most likely the ESCRT complexes, are not involved in miR-451a exosomal sorting. Consistent with the previous observation that miRNAs can be sorted to exosomes through a ceramide-dependent mechanism [4], treating cells with GW4869, an inhibitor of neutral sphingomyelinase 2 regulating ceramide biosynthesis, decreased exosomal miR-451a secretion (Fig 8C). Our data suggest that miR-451a exosomal sorting is regulated by ceramide but not ESCRT.

## Discussion

Here we show that the cytosolic RNA-binding protein MEX3C interacts with the AP-2 complex and associates with the endolysosome system. All three MEX3C variants (MEX3C-1, MEX3C-2, and MEX3C-3) can associate with AP-2, and the interaction is RNA independent: 1) RNase treatment eliminated MEX3C/THOC4 interaction but not MEX3C/AP-2 interaction; 2) The C-terminal truncated MEX3C mutant (MEX3C-1ΔC) preserved RNA binding and nuclear export activity [28] but lost the ability to interact with AP-2 (this study); 3) MEX3C-1mKH mutant lost RNA binding activity [25,26] yet still interacted with AP-2 (this study).

The MEX3C/AP-2 interaction differs in several aspects from AP-2’s interaction with its typical membrane cargos. First, multiple regions are needed for MEX3C to interact with AP-2: a region spanning the KH domain shared by all three MEX3C variants and the C-terminal ring finger domain with ubiquitin E3 ligase activity. Although the YXXΦ motif is important for membrane cargos to associate with AP-2 [18], the two YXXΦ-like motifs of MEX3C do not contribute to MEX3C/AP-2 interaction. This observation is consistent with data showing that the AP-2 YXXΦ binding surface is inaccessible to non-membrane proteins [46].

Ubiquitin modification is a signal to internalize membrane proteins through endocytosis. MEX3C’s ring finger domain with ubiquitin E3 ligase activity is necessary for its interaction with AP-2. Since MEX3C-KO mutants (unable to be ubiquitinated) could interact with AP-2, ubiquitination of MEX3C itself is not necessary for the interaction. However, replacing the ring finger domain by one or two ubiquitin chains rescued the interaction. These seemingly contradictory observations could be explained if we presume that MEX3C’s ring finger domain ubiquitinates an unknown substrate which still binds to MEX3C after ubiquitin modification. In this case, the ubiquitin chain fused to MEX3C substitutes the ubiquitin chain added to the MEX3C-interacting protein by MEX3C’s ring finger domain, enabling MEX3C/AP-2 interaction. Our result is consistent with recent observations of ubiquitination as a mechanism to transport soluble mycobacterial and eukaryotic proteins to the exosomes [47]. The ubiquitin adaptors ESP15 and EPSIN recognize and bind ubiquitin [24], and ESP15 and EPSIN both interact with AP-2 [21–23]. It is possible that for non-membrane proteins such as MEX3C, more than one signal is necessary for their being recruited to the endolysosome system. In the case of MEX3C, the region spanning the KH domain shared by all three variants and the ring finger domain with the ubiquitin E3 ligase activity are both necessary for its interaction with AP-2. While the role of the shared KH-spanning region in this interaction is unclear, the ring finger domain may serve to ubiquitinate an unidentified MEX3C-interaction partner. With the help of ubiquitin adaptors ESP15 or EPSIN, the otherwise weak MEX3C/AP-2 interaction is reinforced (Fig 8D).

The MEX3C/AP-2 interaction may provide a mechanism for MEX3C to associate with the endolysosomal system. Due to the highly dynamic nature of membrane trafficking, transiently expressed MEX3C showed little co-localization with wild-type dynamin. However, when pinching of clathrin-coated vesicles was blocked by mutant dynamin^K44A^, MEX3C was trapped at the dynamin^K44A^-enriched foci, a process depending on AP-2 and the MEX3C domains necessary for MEX3C/AP-2 interaction. MEX3C’s association with the endolysosomal system is also supported by observations that (a) MEX3C degradation was inhibited by the lysosome acidification inhibitor Bafilomycin A1, (b) a single ubiquitin chain decreased MEX3C expression through a post-transcription mechanism, and (c) MEX3C co-localized with AGO2, a protein interacting with MEX3C [25] and associated with endosomes [37,38]. Lack of cytosolic MEX3C-2 in MEX3C-2 and CD63 double-positive cells could also be the result of enhanced sorting of MEX3C-2 to the lysosome for degradation, due to overexpression of CD63, a protein involved in sorting in the multivesicular bodies [48].

The association of RNA-binding protein MEX3C with the endolysosomal system through AP-2 may provide a mechanism for the RNA-endosome association. MEX3C may recruit its direct RNA targets (through its KH domains) to the endolysosomal system. It may also recruit RNAs to the endolysosomal system indirectly through associating with other RNA-binding proteins. AGO2, which interacts with MEX3C on one hand [25], and associates with miRNAs on the other, is a good candidate. We find that another RNA-binding protein, DHX9 (also called RHA), was also pulled down by MEX3C. Thus MEX3C may serve as an adaptor for the recruitment of a variety of RNA species to the endolysosomal compartment. We recently reported that MEX3C-1 is an adaptor for exportin 1-mediated *FOS* mRNA nuclear export [28]. The present work suggests that all MEX3C variants may serve as adaptors for RNA-vesicle association in the cytoplasm.

Inhibiting MEX3C or AP-2 expression decreased exosomal miR-451a expression. This observation supports MEX3C’s role as an adaptor between RNA and the endolysosomal compartment. In addition, it may provide a mechanism for miRNA exosomal sorting. Some miRNAs are sorted to the exosomes in a ceramide-mediated process [3,4]. Agreeing with these observations, inhibiting ceramide synthesis by GW4869 decreased miR-451a exosomal expression. MEX3C and AP-2 may act to increase the association of miR-451a with the endosome, especially the multivesicular bodies generating exosomes. Thus, more miR-451a could be sorted into the intraluminal vesicles through a ceramide-mediated process (Fig 8E). Although miR-451a is not a direct RNA target of MEX3C, other RNA-binding proteins, for example AGO2, could provide a bridge between MEX3C and miR-451a. Since inhibiting MEX3C or AP-2 decreased exosomal but not cellular miR-451a, MEX3C and AP-2 are most likely involved in exosomal sorting but not biogenesis of miR-451a. It is technically challenging to determine whether AGO2 is involved in miR-451a exosomal sorting, since AGO2 is also involved in miR-451a biogenesis. Further work is needed to determine the possible involvement of AGO2 in miR-451a exosomal sorting.

Although miR-451a was the only miRNA affected by MEX3C or AP-2 inhibition in this study, other unidentified RNAs could also be affected. Our analyses of miR-146a-5p and miR-150-5p were inconclusive due to their low exosomal expression in HEK293T cells. Since we have little information on the percentage of total miR-451a secreted into the exosomes, it is unclear whether MEX3C or AP-2 inhibition affects miR-451a transcription or biogenesis. Since miR-16-5p is much more abundant than miR-451a in HEK293T EVs (the average threshold cycle numbers were 16 and 21 respectively), decrease of exosomal miR-451a would have little effect on the amount of total exosomal RNA. Thus, it is likely that MEX3C or AP-2 knockdown affects a subset of EV miRNA.

Multiple studies suggest the involvement of membrane trafficking in RNA transport and localization: the association of fungi septin mRNA with endosomes [49], the interaction of endosome-associated Upa1 with RNA-binding protein Rrm4 [50] which mediates long-distance transport of ubi1 and rho3 mRNAs [51], the requirement of Rab11 for transport of osk mRNA in Drosophila oocytes [52], and the involvement of ESCRT-II complex in RNA transport in Drosophila [53] and *Xenopus* [54]. However, how RNA associates with the membrane system is unknown in most cases. MEX3C/AP-2 interaction may provide a mechanism in mammalian cells for the association of RNA molecules with membrane vesicles.

*In vitro* pull down assays are necessary to examine whether the AP-2 and MEX3C interaction is direct or indirect. However, our finding that the MEX3C and AP-2 interaction depends on multiple elements prevents us from performing such assays. Even though MEX3C and AP-2 may have direct contacts in the complex, we cannot observe direct interactions between the two without additional unidentified components. Nevertheless, our finding of MEX3C/AP-2 association, whether direct or indirect, provides a mechanism for RNA-membrane association. In addition, the MEX3C/AP-2 interaction may increase miR-451a exosomal sorting by increasing the association of miR-451a to the endosome.

## Supporting information

S1 File Supplementary data and information. **Fig A**. MEX3C proteins were enriched in Dynamin^K44A^ mutant positive foci. **Fig B**. MEX3C showed little co-localization with RAB5A, RAB11, LAMP1, and ER marker. **Fig C**. Bafilomycin A1 inhibited MEX3C-1 degradation. **Fig D**. MEX3C-1 did not co-localize with GW182. Fig E. MEX3C-1 pulled down *FOS* mRNA but not miR-451a (n = 2). **Table A**. Plasmids used in the present study. **Table B**. Forward primers used for quantitative PCR analysis of miRNA. **Table C**. EV concentration and size distribution after MEX3C or AP-2 inhibition. **Table D**. Exosome concentration and size distribution after MEX3C inhibition.
(DOCX)
