# Supplementary material for: MEX3C interacts with adaptor-related protein complex 2 and involves in miR-451a exosomal sorting
Source: PLoS One. 2017 Oct 5;12(10):e0185992. doi: 10.1371/journal.pone.0185992 (PMC5628917; doi:10.1371/journal.pone.0185992)
Supplement: S1 File — Fig A. MEX3C proteins were enriched in DynaminK44A mutant positive foci. Fig B. MEX3C showed little co-localization with RAB5A, RAB11, LAMP1, and ER marker. Fig C. Bafilomycin A1 inhibited MEX3C-1 degradation. Fig D. MEX3C-1 did not co-localize with GW182. Fig E. MEX3C-1 pulled down FOS mRNA but not miR-451a (n = 2). Table A. Plasmids used in the present study. Table B. Forward primers used for quantitative PCR analysis of miRNA. Table C. EV concentration and size distribution after MEX3C or AP-2 inhibition. Table D. Exosome concentration and size distribution after MEX3C inhibition. (DOCX) [file pone.0185992.s001.docx]

**MEX3C interacts with adaptor-related protein complex 2 and involves in miR-451a exosomal sorting**

Pin Lu^1,2^, Huanhuan Li^2^, Ning Li^2^, Ravi N. Singh^3^, Colin E. Bishop^2^, Xiangxian Chen^1,2^*, Baisong Lu^2^*

^1^Anhui Normal University, Wuhu 241000, China

^2^Institute for Regenerative Medicine, Wake Forest University Health Sciences, Winston Salem, NC, 27157

^3^Department of Cancer Biology, Wake Forest University Health Sciences, Winston-Salem, NC, 27157

**
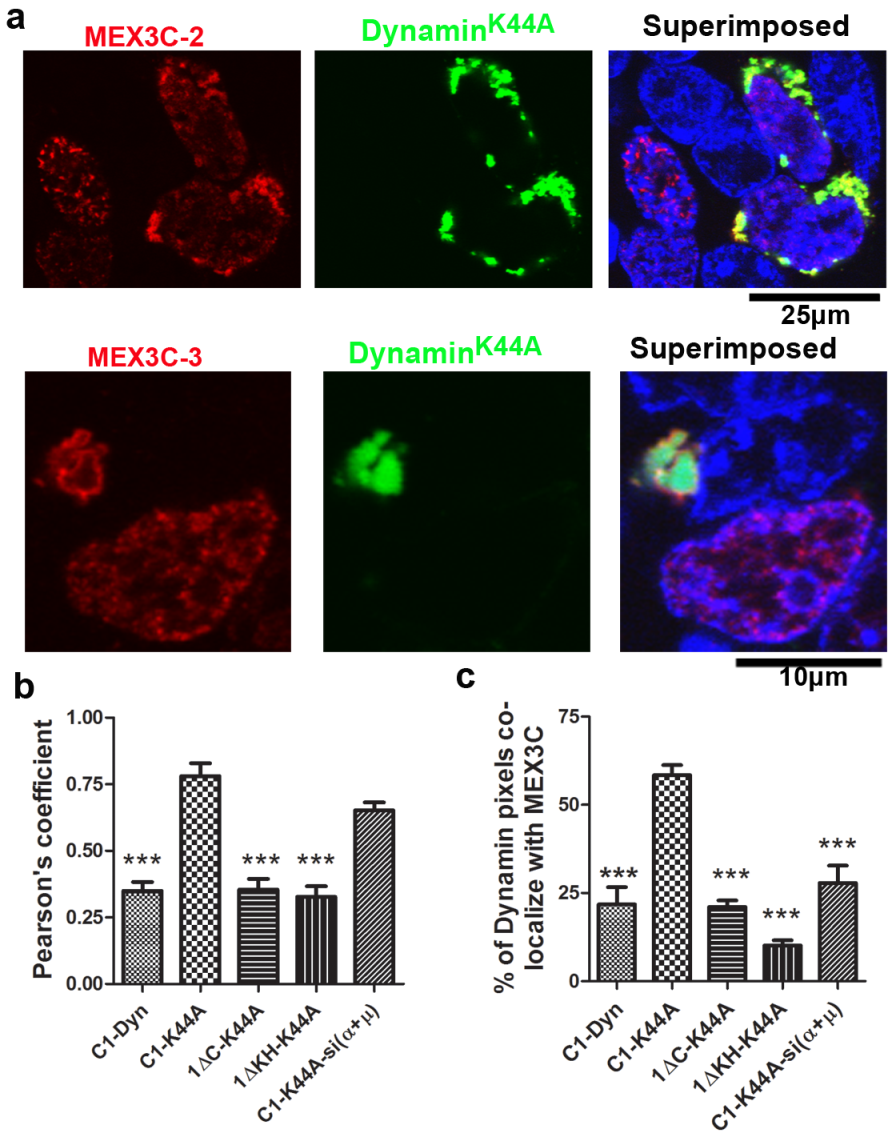
**

**Fig A**. MEX3C proteins were enriched in Dynamin^K44A^ mutant positive foci. **a**. MEX3C-2 and MEX3C-3 were enriched in Dynamin^K44A^-positive foci. MEX3C-2 and MEX3C-3 were Flag-tagged, and Dynamin^K44A^ was GFP tagged. Only cytoplasmic MEX3C-2 was enriched in Dynamin^K44A^-positive foci. Nuclear MEX3C-3 was visible in Dynamin^K44A^-negative cells, while in Dynamin^K44A^-positive cells all MEX3C-3 was cytoplasmic and co-localized with Dynamin^K44A^. **b**. Comparison of Pearson’s correlation coefficient of various co-localization. C1: MEX3C-1; Dyn: wild type Dynamin; K44A: Dynamin^K44A^ mutant; 1∆C: MEX3C-1-∆C; 1∆KH: MEX3C-1-∆KH; si(α+μ): the α and μ2 subunits of AP-2 were inhibited by siRNAs. **C**. Percentage of dynamin-positive pixels co-localized with MEX3C-1. For **b** and **c**, 10 to 20 double-positive cells were analyzed for each condition. Means ± S.E.M are shown. *** indicates p<0.0001 by Tukey’s Post Tests following ANOVA.


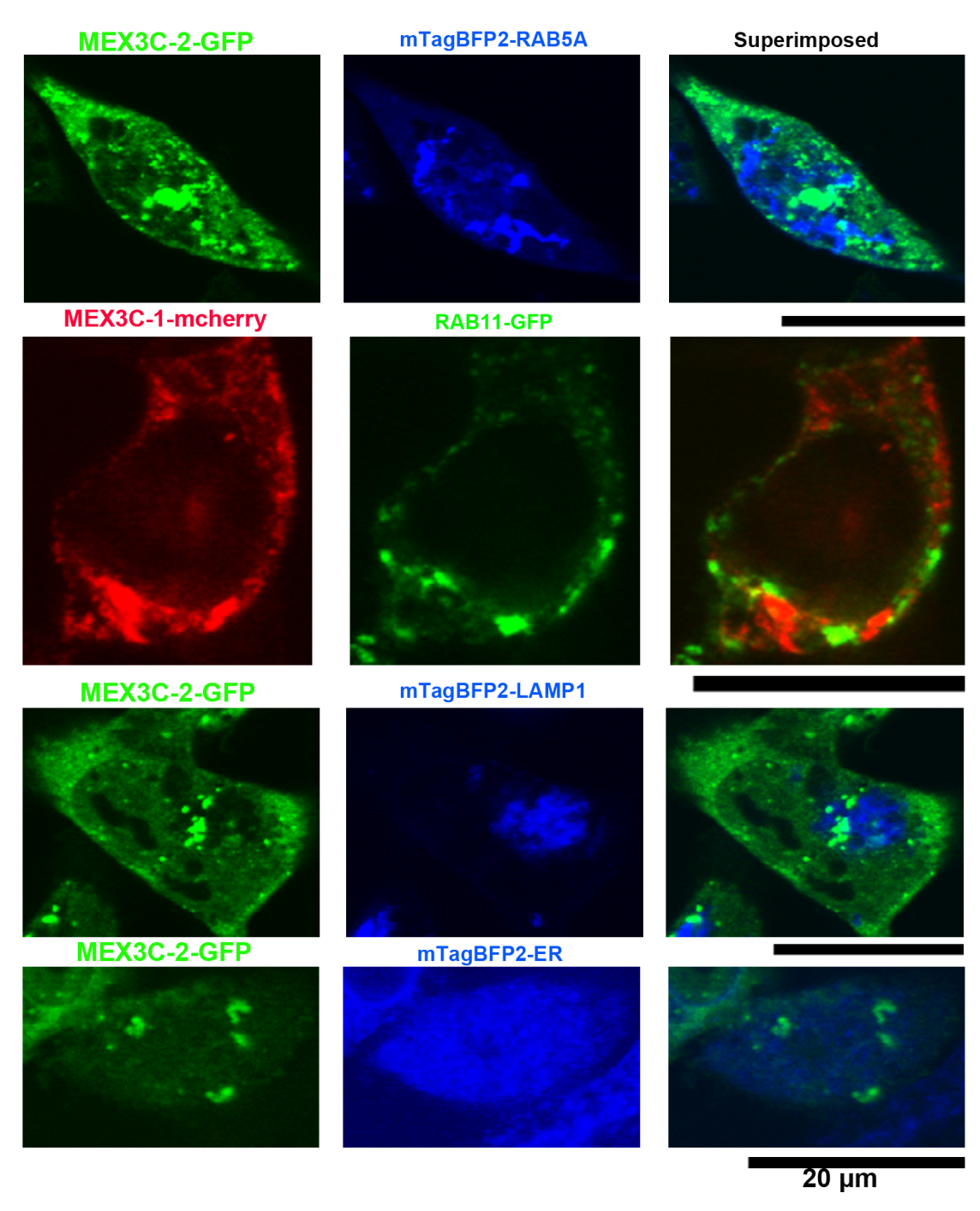


**Fig B**. MEX3C showed little co-localization with RAB5A, RAB11, LAMP1 and ER marker. MEX3C-2-GFP and RAB11-GFP were pseudocolored green. MEX3C-1-mCherry was pseudocolored red. mTagBFP2-LAMP1, mTagBFP2-ER and mTagBFP2-RAB5A were pseudocolored blue.


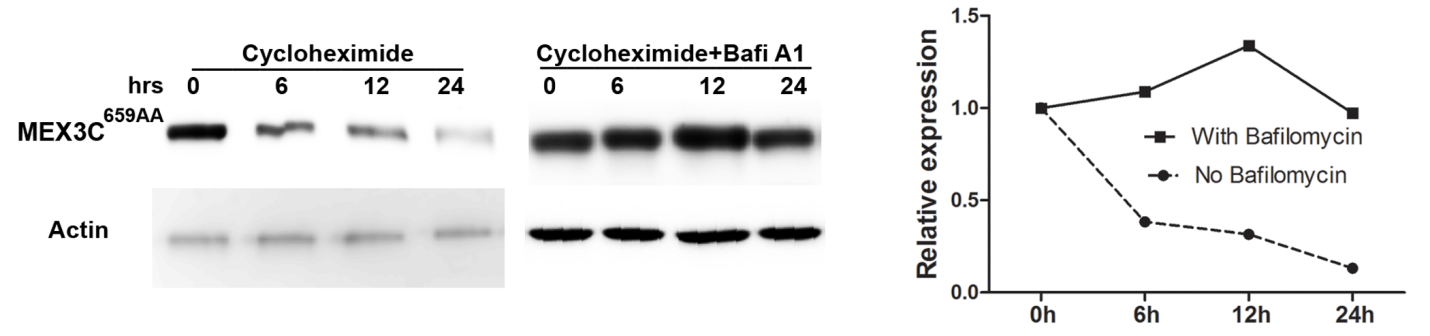


**Fig C**. Bafilomycin A1 inhibited MEX3C-1 degradation. Protein translation was inhibited by cycloheximide, in the presence and absence of Bafilomycin A1. MEX3C-1 was detected by Western blotting at various time points after protein translation inhibition. Relative expression was determined by densitometry after loading control normalization.

**
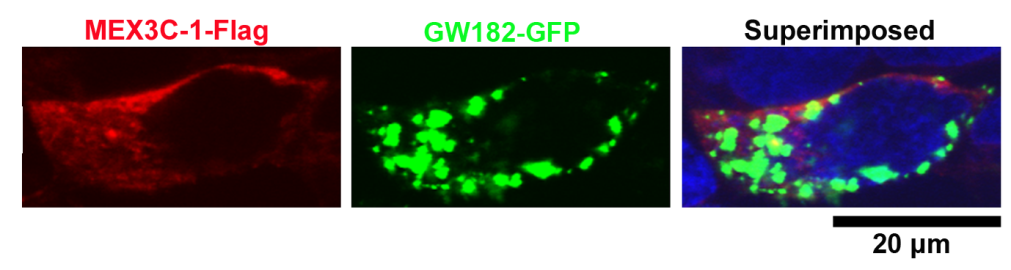
**

**Fig D**. MEX3C-1 did not co-localize with GW182. MEX3C-1 was Flag-tagged and GW182 was GFP-tagged. The similarity of GW182-GFP to endogenous GW182 was validated by the donating investigator. Shown are representative images of multiple double-positive cells examined. Nuclei (stained by DAPI) were pseudocolored blue.

**
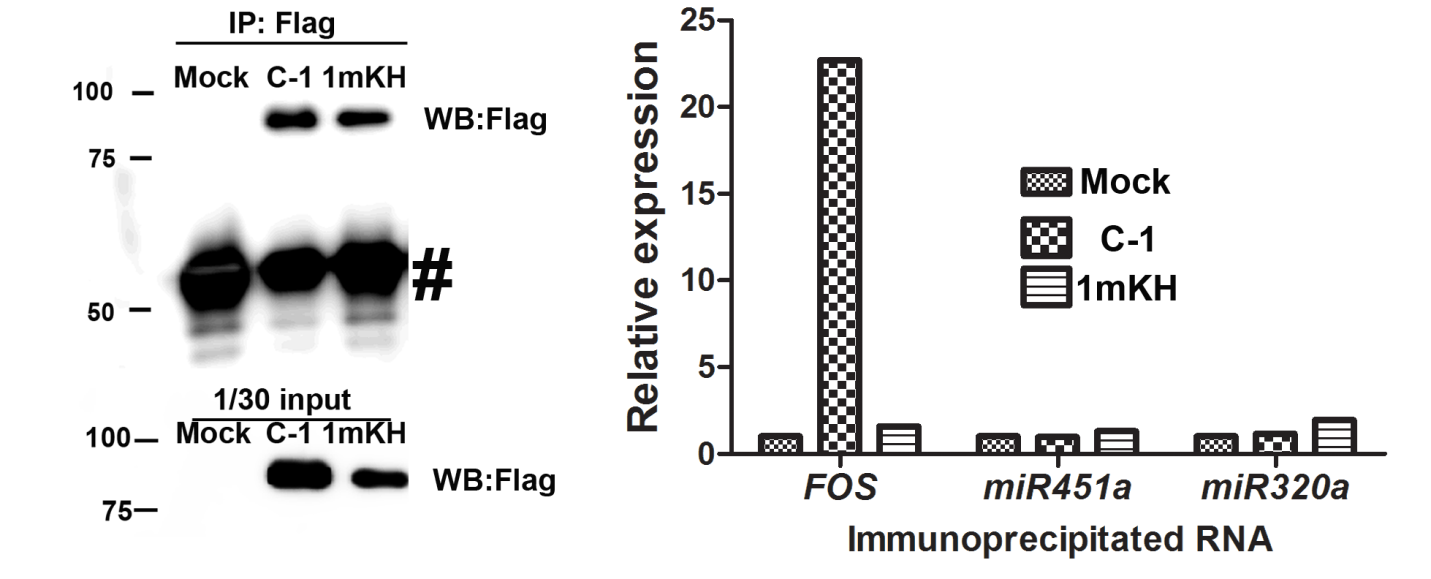
**

**Fig E**. MEX3C-1 pulled down *FOS* mRNA but not miR-451a (n=2). Left: Western blotting showed that the Flag-tagged MEX3C-1 and MEX3C-1-mKH were successfully immunoprecipitated. # indicates the IgG bands. Right: Real time RT-PCR analysis of RNAs pulled down by MEX3C-1 and MEX3C-1-mKH.

**Table A. Plasmids used in the present study**

| **Name** | **Purpose** | **Construction strategy** |
| --- | --- | --- |
| pRK5-MEX3C-2-KO-HA | Expressing mutated HA-tagged MEX3C-2 (MEX3C-2-KO-HA), all lysine residues were mutated to arginine. | Full-length cDNAs coding for MEX3C-2-KO-HA was synthesized and inserted into the *BamH1* and *SalI* sites of pRK5F. |
| pRK5-MEX3C-3-KO-HA | Expressing mutated HA-tagged MEX3C-3 (MEX3C-3-KO-HA), all lysine residues were mutated to arginine. | Full-length cDNAs coding for MEX3C-3-KO-HA was synthesized and inserted into the *BamH1* and *SalI* sites of pRK5F. |
| pRK5-MEX3C-1-KO-HA | Expressing mutated HA-tagged MEX3C-1 (MEX3C-1-KO-HA), all lysine residues were mutated to arginine. | Amplified by PCR the cDNA encoding human MEX3C-1 amino acids 1-195 from pFlag-MEX3C-1 with primers *659KO-F* (AGGATCCACCATGCCTTCTGGGTCATCAGCCGCA) and *659KO-R* (AGGATCCCTGAGCATCGTCTCCCCCGTAC), then inserted the cDNA into the *BamH1* site of pRK5-MEX3C-2-KO-HA. |
| pRK5-MEX3C-2-KO-Flag | Expressing Flag-tagged mouse MEX3C-2-KO (pRK5-MEX3C-2-KO-Flag) | Replacing the AleI-SalI fragment of pRK5-MEX3C-2-KO-HA with a synthesized AleI-SalI fragment, which had the Flag tag coding sequence instead of the HA tag coding sequence |
| pFlag-MEX3C-2-mRing | Expressing the Flag-tagged, Ring finger mutated mouse MEX3C-2 (Flag-MEX3C-2-mRing). | Replacing the *AleI*-*HindIII* fragment of pFlag-MEX3C-2 with the PCR fragment amplified from the same template with primers PHD-F (acacgacAgtgtgattAgctttgagaatgaggttattgctgccctagttccaAgtggcAacaac, capital letters indicate those mutating the Ring finger domain) and rkhd2R (aatgtcgacAGAGTGAATTTGGATTGCCTGAG). The Cys(3)-His-Cys(4) sequence important for the E3 ligase activity was changed to Ana(3)-Asn-Cys(4) |
| pFlag-MEX3C-1mY | Expressing the tyrosine-based Yxxψ sorting signal-mutated MEX3C-1 (Flag-MEX3C-1mY) | Replacing the EcoO109I-EcoRV fragment of pFlag-MEX3C-1 with a synthesized EcoO109I-EcoRV fragment, where ^335^YRVV were mutated to ^335^ARVA, and ^398^YIEL were mutated to ^398^AIEA. |
| pFlag-MEX3C-1mKH | Expressing the KH domains mutated MEX3C-1 (Flag-MEX3C-1mKH) | Replacing the BmgBI-EcoRI fragment of pFlag-MEX3C-1 with a synthesized BmgBI-EcoRI fragment, where ^249^Gly and ^343^Gly, two residues important for RNA binding [[25](#_ENREF_25),[26](#_ENREF_26)] were mutated to Asp. |
| pMEX3C-1KO-∆Ring-UbKO | Expressing mutant MEX3C-1KO-∆Ring-UbKO, all lysine residues of MEX3C-1 were mutated to arginine, the C-terminal 53AA Ring finger domain was deleted, the truncated MEX3C mutant was then fused with a mutated ubiquitin where all lysine residues were mutated to arginine. | Inserted the cDNA coding for HA-UbKO [amplified from pRK5-HA-UbKO (purchased from Addgene) with primers Ale-F ( AAGAAGAAGGCACGAcGGTGGAATGGGCTACCCCTATGATGTG) and Ale-R (AAGCAAATCACACAGTCAACCACCTCTTAGTCTTAAGAC)] into the AleI site of pMEX3C-1-KO-HA by In-Fusion cloning (Clonetech). |
| pMEX3C-1KO-∆Ring-2xUbKO | Expressing mutant MEX3C-1KO-∆Ring-2xUbKO, all lysine residues of MEX3C-1 were mutated to arginine, the C-terminal 53AA Ring finger domain was deleted, the truncated MEX3C mutant was then fused with two mutated ubiquitin where all lysine residues were mutated to arginine. | Inserting the cDNA coding for HA-UbKO [amplified from pRK5-HA-UbKO with primers Ale-F and Ale-R1 (TAGCCCATTCCACCggAACCACCTCTTAGTCTTAAGAC)] into the AleI site of pMEX3C-1-KO-∆ring-UbKO by In-Fusion cloning. |
| pMEX3C-1-KO-∆Ring |  | Cut pMEX3C-1-KO-∆ring-UbKO with *SalI* to remove the sequences coding for ubiquitin, and then self-ligated the remaining backbone. |
| mTagBFP2-Rab5a-7 |  | Addgene plasmid # 55322 |
| mTagBFP2-Lysosomes-20 |  | Addgene plasmid # 55308 |
| mTagBFP2-ER-5 |  | Addgene plasmid # 55294 |
| mTagBFP2-TOMM20-N-10 |  | Addgene plasmid # 55328 |
| EGFP-Rab11a-7 |  | Addgene plasmid # 56444 |
| mAzurite-Clathrin-15 |  | Addgene plasmid # 55228 |
| Wt dynamin 1 pEGFP |  | Addgene plasmid # 34680 |
| K44A dynamin 1 pEGFP |  | Addgene plasmid # 34681 |
| psigma2-EGFP |  | Addgene plasmid # 53610 |
| AP2u2-mCherry |  | Addgene plasmid # 27672 |
| CD63-pEGFP C2 |  | Addgene plasmid # 62964 |

**Table B. Forward primers used for miRNA quantitative PCR analysis**

| miRNA | Forward primer sequence |
| --- | --- |
| miR-150-5p | TCTCCCAACCCTTGTACCAGTG |
| miR-146a-5p | TGAGAACTGAATTCCATGGGTT |
| miR-451a | AAACCGTTACCATTACTGAGTT |
| miR-16-5p | TAGCAGCACGTAAATATTGGCG |
| miR-320a | AAAAGCTGGGTTGAGAGGGCGA |
| let-7a-5p | TGAGGTAGTAGGTTGTATAGTT |
| miR-39-3p | TCACCGGGTGTAAATCAGCTTG |

**Table C. EV concentration and size distribution after MEX3C or AP-2 inhibition**

| **Treatment group** | **Ave EV concentration**  **(10^10^ particles/ml)^a^** | **Ave EV size (nm)** |
| --- | --- | --- |
| ***si-Luci*** | 1.31 ± 0.166 | 185.30 ± 1.8 |
| ***si-MEX3C*** | 2.43 ± 0.069 | 191.90 ± 2.0 |
| ***si-AP-2 α & μ*** | 4.36 ± 0.208 | 195.20 ± 1.1 |

^a^Mean ± S.E.M. EVs were collected from serum-free supernatant of 1x10^7^ cells during 24-96 hours after siRNA transfection. The EVs were re-suspended in 1 ml PBS for Nanosight analysis.

**Table D. Exosome concentration and size distribution after MEX3C inhibition**

| **Treatment group** | **Ave EV concentration**  **(10^8^ particles/ml)^a^** | **Ave EV size (nm)** |
| --- | --- | --- |
| ***Non-targeting control*** | 1.16 ± 0.355 | 85.30 ± 21.5 |
| ***shRNA-coding*** | 0.917 ± 0.141 | 117.90 ± 30.2 |
| ***shRNA-UTR*** | 0.999 ± 0.319 | 122.65 ± 30 |

^a^Mean ± S.E.M. Exosomes were collected from serum-free supernatant cultured with 1x10^7^ cells for 3 days. Before addition of serum-free medium, the cells have been induced by 1 μg/ml DOX for 3 days. The exosomes were re-suspended in 1 ml PBS for analysis.
